# A multifunctional sesquiterpene synthase integrates with cytochrome P450s to reinforce the terpenoid defense network in maize

**DOI:** 10.1111/tpj.70575

**Published:** 2025-11-14

**Authors:** Mengxi Wu, Tobias G. Köllner, Elly Poretsky, Zhouxin Shen, Steven P. Briggs, Alisa Huffaker, Eric A. Schmelz, Yezhang Ding

**Affiliations:** ^1^ Department of Cell and Developmental Biology, School of Biological Sciences University of California at San Diego La Jolla California 92093 USA; ^2^ Department of Natural Product Biosynthesis Max Planck Institute for Chemical Ecology Jena D‐07745 Germany; ^3^ Present address: College of Landscape Architecture Sichuan Agricultural University Chengdu 611130 P.R. China; ^4^ Present address: Environmental Genomics and Systems Biology Division Lawrence Berkeley National Laboratory Berkeley California 94720 USA

**Keywords:** maize specialized metabolism, sesquiterpenoids, terpene synthase, α‐santalenoic acid, cytochrome P450, defense network

## Abstract

Terpenoids, the largest and most structurally diverse class of plant natural products, play essential roles in maize defense and ecological interactions. In this study, we identified and functionally characterized a sesquiterpenoid‐based defense pathway in maize centered on α‐santalenoic acid, a pathogen‐inducible sesquiterpenoid antibiotic. Using a combination of metabolite‐based genome‐wide association studies (mGWAS), linkage mapping, and heterologous expression assays, we identified ZmTPS9 as a multiproduct terpene synthase that primarily produces α‐santalene and β‐bisabolene. Sequence analysis and site‐directed mutagenesis revealed that threonine at position 413 is critical for enzyme activity, with its deletion resulting in a complete loss of enzyme activity. The sesquiterpene hydrocarbons produced by ZmTPS9 are further oxidized by three cytochrome P450 monooxygenases, ZmCYP71Z16, ZmCYP71Z18, and ZmCYP71Z19, to yield antimicrobial metabolites including α‐santalenoic acid, zealexin D1 (ZD1), and zealexin D2 (ZD2). Together, these findings demonstrate a convergent biosynthetic strategy in maize, where multiproduct terpene synthases and promiscuous P450s collaboratively generate a flexible and robust terpenoid defense network.

## Significance Statement

This study uncovers a previously unknown maize sesquiterpenoid defense pathway centered on the antibiotic α‐santalenoic acid. Using integrated genetic and biochemical approaches, we identified ZmTPS9 as a pathogen‐inducible terpene synthase responsible for producing multiple sesquiterpenes, primarily α‐santalene and β‐bisabolene. These olefin precursors are further oxidized by three cytochrome P450 enzymes into bioactive metabolites, including α‐santalenoic acid and zealexins. Our findings highlight a convergent biosynthetic strategy underpinning maize's versatile and robust terpenoid defense network.

## INTRODUCTION

Continuous genetic evolution leads to the development of diverse natural products in plants that play important roles in plant defense against both biotic and abiotic stresses (Jia et al., 2022; Pichersky & Gang, 2000). Terpenoids, the largest and most diverse class of plant natural products, contribute significantly to the biochemical complexity and diversity of the plant kingdom (Pichersky & Raguso, 2018; Zi et al., 2014). The majority of terpenoids are involved in plant interactions with their environment, such as attracting pollinators, repelling herbivores, and defending against pathogens as well as environmental stresses (Desmedt et al., 2022; Ding et al., 2021; Erb & Kliebenstein, 2020). Sesquiterpenoids constitute a highly abundant and structurally diverse class of terpenoids that are broadly distributed across higher plants and display a wide spectrum of biological activities (Lange & Ahkami, 2013). Among them, sesquiterpenoid phytoalexins such as zealexins, α/β‐costic acids, gossypol, capsidiol, and rishitin exhibit antifungal activity against plant pathogens (Bulasag et al., 2024; Ding et al., 2017; Huffaker et al., 2011). Beyond their defensive roles, sesquiterpenoids have significant industrial applications in fragrances, flavors, and medicine, as exemplified by the well‐known sesquiterpenoids, artemisinin, bisabolol, and santalol (Celedon et al., 2016; Fraga, 2013). Despite notable advances, our understanding of sesquiterpenoid diversity, associated metabolites, and the genes underlying their biosynthetic pathways remains incomplete. Expanding this knowledge will be critical for developing new strategies to enhance crop resilience under increasingly variable environmental conditions (Ding et al., 2021; Yasmin et al., 2024).

Originating from a C15 precursor, farnesyl diphosphate (FPP), plant sesquiterpenoids are typically produced through the sequential activity of enzymes encoded by two functionally diverse gene families: terpene synthases (TPSs) and cytochrome P450 monooxygenases (P450s) (Mafu et al., 2018; Wang & Peters, 2023). TPSs act as gatekeepers in the generation of terpenoids by catalyzing the conversion of prenyl diphosphates with varying chain lengths or different *cis/trans* configurations as substrates to diverse terpene skeletons (Karunanithi & Zerbe, 2019). Interestingly, there is tremendous catalytic promiscuity in terpene synthases, often resulting in the production of multiple terpenes by a single TPS enzyme (Beran et al., 2019; Luck et al., 2020). An example in maize is ZmTPS4, which converts FPP into a complex blend of sesquiterpenes, with 7‐*epi*‐sesquithujene and β‐bisabolene being the major products (Köllner et al., 2006). SaSSy, a sesquiterpene synthase from sandalwood (*Santalum album*), was also found to produce a mixture consisting of α‐*endo*‐bergamotene, α‐santalene, and β‐santalene (Jones et al., 2011). Likewise, CmCJTPS3 from *Chrysanthemum morifolium* was capable of catalyzing the formation of α‐bisabolol and a few other sesquiterpenoids, with FPP as the substrate (Zhang et al., 2022). This prevalent trait of TPS product promiscuity greatly amplifies the chemical diversity of terpenoids from a limited number of genes. Furthermore, terpene synthases also exhibit strong functional plasticity, where minor alterations in the active site can significantly affect product outcomes and lead to the evolution of new enzymes with minimal investment (Di Girolamo et al., 2020; Karunanithi & Zerbe, 2019). For example, a single amino acid substitution in the monoterpene synthase CiCaMS from *Cinnamomum camphora* enlarged the active site cavity, enabling the enzyme to accommodate the larger farnesyl diphosphate substrate and facilitate sesquiterpene production (Di Girolamo et al., 2020). P450s are also exceptionally versatile catalysts that can introduce diverse oxygen‐containing functional groups (hydroxyl, epoxide, carbonyl, etc.) or perform other modifications on terpene backbones (Mafu et al., 2018; Wang & Peters, 2023). This “decoration” of terpene scaffolds vastly increases the structural complexity of terpenoids and is frequently critical for their defensive function (Banerjee & Hamberger, 2018; Weitzel & Simonsen, 2013). In maize, terpenoid defense pathways do not follow simple linear routes but instead operate as modular biosynthetic networks in which multiple TPSs and P450s interact combinatorially. For example, the class II diterpene synthase ZmAN2 generates ent‐copalyl diphosphate (ent‐CPP), which is cyclized by ZmKSL2/3 or ZmKSL4 to form distinct diterpene scaffolds. These intermediates are then oxidized by ZmCYP71Z16/18 and additional tailoring enzymes to yield kauralexins and dolabralexins, key phytoalexins that reinforce disease resistance (Ding et al., 2019; Murphy et al., 2023). ZmCYP71Z16 and ZmCYP71Z18 also participate in sesquiterpenoid metabolism, catalyzing stepwise oxidations of β‐bisabolene at C1 and C15 to produce zealexin D1 (ZD1) and zealexin D2 (ZD2), and oxidizing β‐macrocarpene at C15 to form zealexin A1 (ZA1) (Ding et al., 2020). Similarly, ZmCYP71Z19 exhibits broad substrate scope, acting on multiple sesquiterpene olefins in the biosynthesis of zealexins and α/β‐costic acids (Ding et al., 2017, 2020).

In our continuing search for phytochemicals in maize, a detailed investigation of the metabolic profile of maize plants in response to fungal elicitation resulted in the discovery of α‐santalenoic acid, a sesquiterpene carboxylic acid exhibiting natural variation across diverse maize genetic lines and antifungal activity *in vitro*. To elucidate the biosynthetic pathway of α‐santalenoic acid, linkage and association mapping analyses were performed, identifying *ZmTPS9* as a candidate gene. The functionality of ZmTPS9 was further confirmed by *in vitro* enzyme assays, demonstrating its ability to generate multiple sesquiterpene products, predominantly α‐santalene and β‐bisabolene. These hydrocarbon olefins can be further modified by three CYP71Zs (CYP71Z16, CYP71Z18, and CYP71Z19) to produce antibiotic compounds such as α‐santalenoic acid, ZD1, and ZD2. Our results demonstrate that ZmTPS9 significantly contributes to the formation of the cocktail of maize terpenoid antibiotics.

## RESULTS

### Pathogen‐inducible production of α‐santalenoic acid as a sesquiterpenoid antibiotic in maize

**Figure 1:**
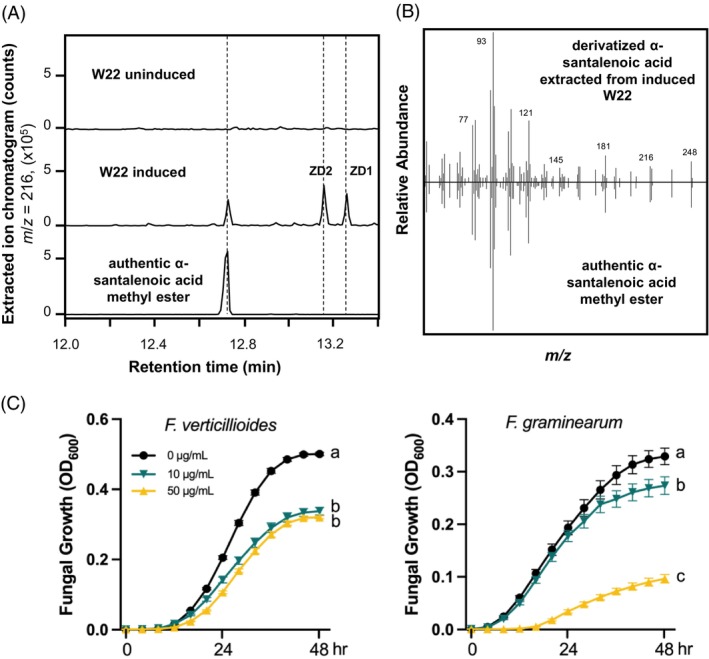
α‐Santalenoic acid functions as an antibiotic against maize fungal pathogens. (A) GC‐MS extracted ion chromatograms (EICs) showing inducible production of α‐santalenoic acid, measured after derivatization as α‐santalenoic acid methyl ester, in W22 stems treated with heat‐killed *F. venenatum* hyphae for 5 days. ZD1, zealexin D1; ZD2, zealexin D2. (B) EI spectra [*m*/*z*] of α‐santalenoic acid methyl ester from plant tissues and a chemical standard. (C) Antifungal activity of α‐santalenoic acid. Average growth (OD_600_) of *F. verticillioides* and *F. graminearum* is shown in the absence and presence of α‐santalenoic acid measured over a 48‐h time course in a defined minimal broth medium using a microtiter plate assay. Error bars represent means ± SEM (*n* = 6), and letters (a–c) represent statistically significant differences at *P* < 0.05 as measured using ANOVA and Tukey's tests to correct for multiple comparisons between control and treatments.

To further investigate terpenoids with potential antifungal activity, we conducted metabolic profiling of maize W22 stem tissues elicited with heat‐killed *Fusarium venenatum* using gas chromatography‐mass spectrometry (GC‐MS). Following elicitation, numerous acidic sesquiterpenoids became prominent in the chromatogram (Ding et al., 2017, 2020). The W22 inbred was chosen because comprehensive omics datasets (RNA‐seq, proteomics, and metabolomics) from fungal‐elicited W22 tissues are available from our previous work (Ding et al., 2019, 2020), as well as well‐established genetic resources. Among the acidic sesquiterpenoids found in the elicited stem tissues, an unidentified analyte was co‐induced with the known sesquiterpene acids, zealexin D1 (ZD1) and zealexin D2 (ZD2) (Figure 1A). The unknown analyte, with a parent ion of 248 *m*/*z*, was subsequently identified as α‐santalenoic acid based on its mass spectrum and retention time in comparison with an authentic standard (Figure 1A,B). α‐Santalenoic acid, a downstream product of α‐santalene, was initially discovered in sandalwood oil (Kamat et al., 1967) and is known to possess significant strong insecticidal properties (Coates et al., 1988; Frelichowski & Juvik, 2001). Furthermore, α‐santalenoic acid has shown antibacterial activity *in vitro* (Xu et al., 2014). To assess the potential antifungal activity of α‐santalenoic acid in maize, *in vitro* assays were performed against two major fungal pathogens, *F. graminearum* and *F. verticillioides*. Fungal growth was measured in nutrient media containing various concentrations of α‐santalenoic acid using a standardized 96‐well microtiter assay (Schmelz et al., 2011). At 10 and 50 μg ml^−1^, α‐santalenoic acid significantly inhibited the growth of both fungal species (Figure 1C), suggesting its role as a pathogen‐inducible antifungal compound in maize.

### Identification of *ZmTPS9* as a candidate biosynthetic gene

**Figure 2:**
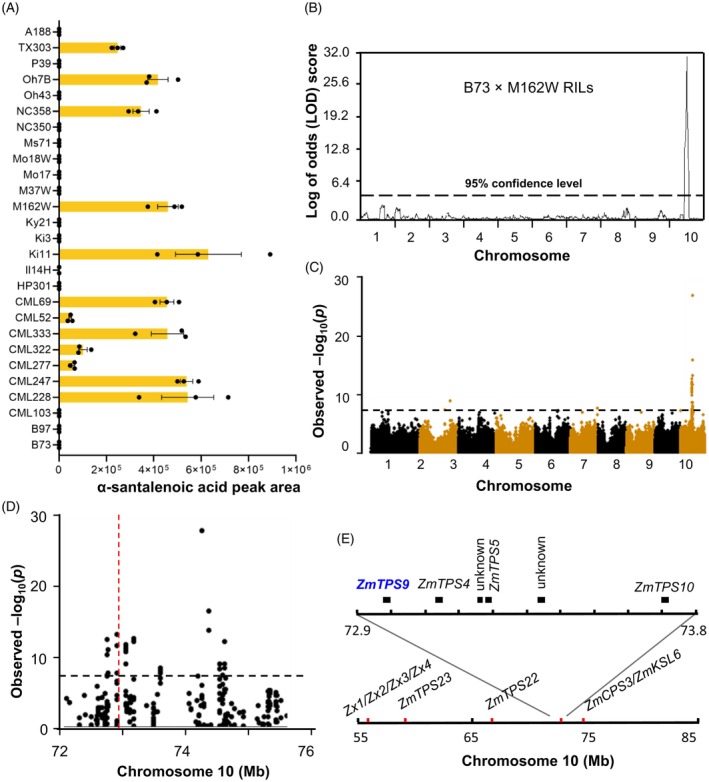
Combined linkage analysis and mGWAS identify ZmTPS9 as a candidate α‐santalene synthase. (A) The levels (peak area, *m*/*z* 216) of α‐santalenoic acid, measured after derivatization as α‐santalenoic acid methyl ester, in the inbred parents of the NAM RILs. The stems of 4‐week‐old inbred lines were treated with heat‐killed *F. venenatum* hyphae for 3 days. Error bars represent means ± SEM (*n* = 3). (B) Linkage analysis of α‐santalenoic acid using the B73 × M162W RIL population. Logarithm of the odds (LOD) score profile with the permutation threshold is shown. cM, centimorgan. (C) Manhattan plot of the Goodman diversity panel association analysis using α‐santalenoic acid as a mapping trait. Negative log_10_ transformed *P* values from the compressed mixed linear model (MLM) are plotted on the *y*‐axis. The dashed line denotes the 5% Bonferroni‐corrected threshold for 246 477 SNP markers. (D) Local Manhattan plot showing the most statistically significant SNPs located between 72 and 76 Mb on chromosome 10 (B73 RefGen_v4). (E) The physical position of the sesquiterpene TPS gene cluster within the mapped locus containing *ZmTPS4*, *ZmTPS5*, *ZmTPS9*, and *ZmTPS10* (B73 RefGen_v4).

Given the potential role of α‐santalenoic acid in maize defense, we sought to identify the gene(s) responsible for its biosynthesis. Fungal‐elicited α‐santalenoic acid was first analyzed in the stems of 27 parental inbred lines previously developed for use in the maize nested association mapping (NAM) populations (Yu et al., 2008). Following fungal elicitation, α‐santalenoic acid accumulation was observed in 12 inbred lines, including M162W, but remained undetectable in 15 lines, such as B73 (Figure 2A). Based on this qualitative variation, the B73 × M162W recombinant inbred line (RIL) population was selected for genetic mapping. Linkage analysis using α‐santalenoic acid abundance as the mapping trait identified a single significant locus on chromosome 10 (Figure 2B). In parallel, a metabolite‐based genome‐wide association study (mGWAS) revealed a cluster of highly significant single‐nucleotide polymorphisms (SNPs) within the same genomic region (Figure 2C), further supporting this locus as a candidate region underlying α‐santalenoic acid biosynthesis. Examination of the B73 reference genome (RefGen v4) revealed four TPS genes within the mapped interval: *ZmTPS4* (*Zm00001d024478*), *ZmTPS5* (*Zm00001d024481*), *ZmTPS9* (*Zm00001d024477*), and *ZmTPS10* (*Zm00001d024486*) (Figure 2D,E) (for maize TPS nomenclature, see Köllner et al., 2023). Of these, *ZmTPS4* and *ZmTPS5* encode multiproduct enzymes that produce bisabolane‐, sesquithujane‐, and bergamotane‐type sesquiterpenes, though with differing product profiles (Köllner et al., 2004, 2006). ZmTPS10 primarily catalyzes the formation of (*E*)‐*β*‐farnesene and (*E*)‐*α*‐bergamotene (Schnee et al., 2006). Therefore, the uncharacterized *ZmTPS9* was finally prioritized as the most likely candidate gene involved in α‐santalenoic acid biosynthesis.

To assess *ZmTPS9* expression in response to fungal challenge, we analyzed our previously published RNA sequencing (RNA‐seq) and mass spectrometry‐based proteomics datasets from W22 stem tissues treated with heat‐killed *F. venenatum* (Ding et al., 2020). Fungal elicitation led to a marked increase in *ZmTPS9* transcript abundance (Figure S1a), exhibiting a similar temporal pattern to other β‐bisabolene/β‐macrocarpene synthase genes (Ding et al., 2020). This transcript‐level induction was paralleled by increased accumulation of ZmTPS9 protein, as detected in a proteomic analysis (Figure S1b). Collectively, these data support a role for ZmTPS9 in maize defense and implicate it as a core component of the α‐santalenoic acid biosynthetic pathway.

### Functional characterization of *ZmTPS9*

**Figure 3:**
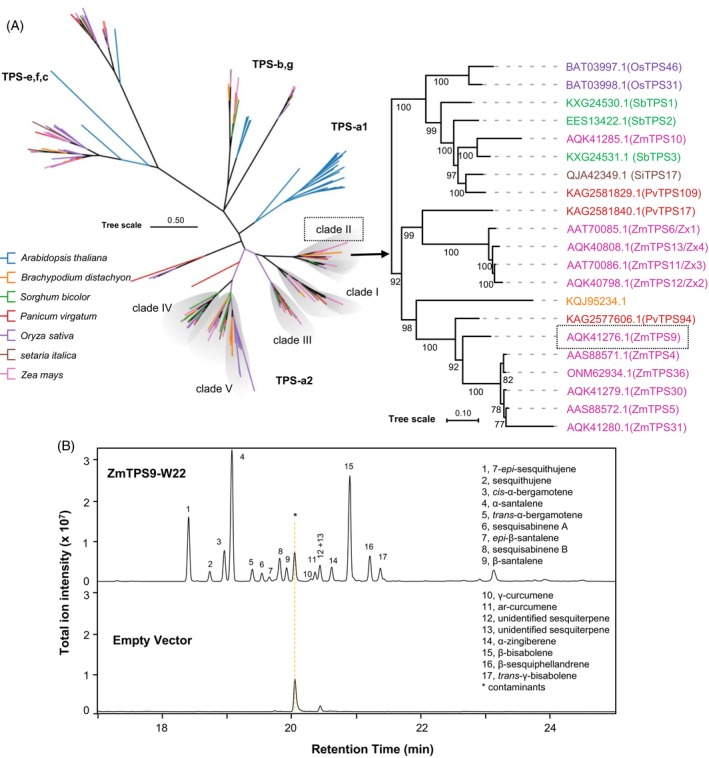
Characterization of *ZmTPS9*. (A) Dendrogram analysis of terpene synthase genes from six Poaceae species (*Oryza sativa* (*Os*), *Sorghum bicolor* (*Sb*), *Zea mays* (*Zm*), *Panicum virgatum* (*Pv*), *Setaria italica* (*Si*), *Brachypodium distachyon* (*Bd*)) and *Arabidopsis thaliana* (*At*). Protein sequences used for analysis were included in Table S6. The phylogenetic tree was constructed using the maximum likelihood method with a bootstrap value of 1000 using IQtree. The best‐fit substitution model was Q.plant+F+R5 according to Bayesian information criterion (BIC) scoring. TPS subfamilies (TPS‐a, TPS‐g, TPS‐b, TPS‐c, and TPS‐e/f clades) are listed according to Luck et al. (2020). The generated phylogenetic tree was further refined using the ChiPlot online tool (https://www.chiplot.online/). (B) Sesquiterpene olefin products of ZmTPS9‐W22. ZmTPS9‐W22 was heterologously expressed in *Escherichia coli*, and the resulting protein extract was incubated with (*E*,*E*)‐FPP. Enzyme products were collected using solid‐phase microextraction (SPME) and analyzed by GC‐MS. Major sesquiterpene products are identified and listed in the figure. A protein extract from *E. coli* expressing an empty vector was used as a negative control.

Phylogenetic analysis classified ZmTPS9 within the TPS‐a2 clade II, a subfamily predominantly composed of sesquiterpene synthases (sesqui‐TPSs) in angiosperms (Luck et al., 2020) (Figure 3A). In contrast, previously characterized santalene‐forming sesqui‐TPSs, such as SaSSy, SauSSy, and SpiSSy, belong to the TPS‐b subfamily, which is mainly associated with monoterpene synthases in flowering plants (Jones et al., 2011). Enzymes in TPS‐a2 clades I–III typically exhibit clade‐specific catalytic characteristics, determined either by substrate specificity or by the nature of the initial cyclization step. Specifically, TPS‐a2 clade II enzymes often generate cyclic sesquiterpenes through a C6–C1 cyclization of farnesyl diphosphate (FPP), or alternatively, produce acyclic sesquiterpenes (Luck et al., 2020).

**Figure 4:**
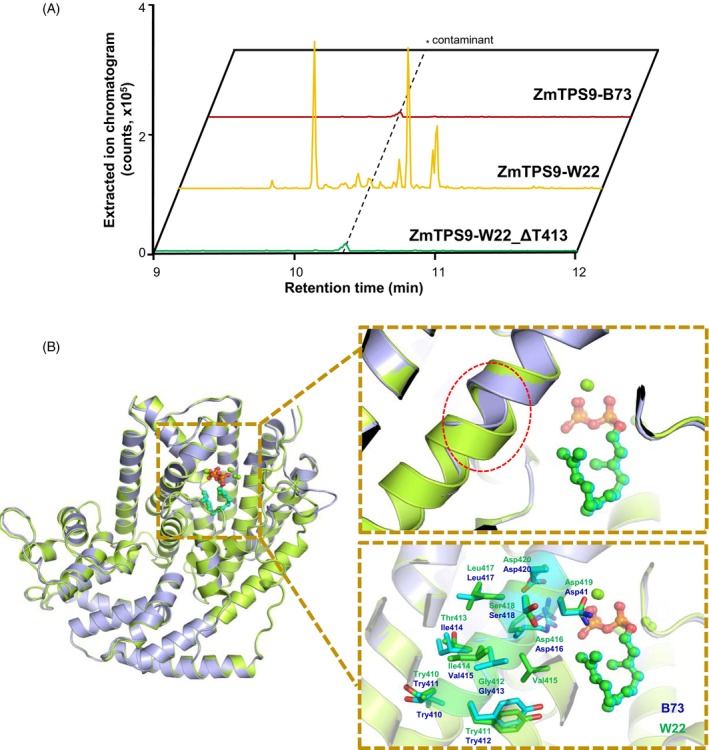
An amino acid deletion abolishes ZmTPS9 enzymatic function. (A) GC‐MS extracted ion chromatograms (EICs) are shown for the volatiles produced by *Escherichia coli* co‐expressing maize farnesyl diphosphate synthase ZmFPS3 with ZmTPS9‐B73, ZmTPS9‐W22, or a threonine‐413 deletion mutant (ΔT413) of ZmTPS9‐W22, respectively. (B) Structural comparison of predicted tertiary models of ZmTPS9 from B73 (purple) and W22 (green), highlighting conformational differences associated with the loss of threonine at position 413.

To evaluate the biochemical activity of ZmTPS9, a full‐length cDNA was isolated from fungal‐elicited W22 stem tissue and heterologously expressed in *Escherichia coli*. The resulting protein extract was incubated with the precursor (*E*,*E*)‐FPP *in vitro*, resulting in the production of a complex mixture of sesquiterpene olefins, with α‐santalene and β‐bisabolene as the predominant products (Figure 3B; Table S1). In addition, co‐expression of *ZmTPS9‐W22* with *ZmFPS3* in *E. coli* and trapping the *E. coli*‐produced volatiles yielded a comparable blend of sesquiterpene olefins (Figure 4A). In contrast, co‐expression of *ZmTPS9* from the maize inbred line B73 (*ZmTPS9‐B73*) with *ZmFPS3* failed to yield detectable terpene products (Figure 4A), indicating that the B73 allele encodes an inactive or nonfunctional enzyme. Functional divergence of terpene synthases, through neofunctionalization or pseudogenization, has also been observed in other plant species, such as tomato and rice (Zhan et al., 2020; Zhou & Pichersky, 2020).

The formation of α‐santalene and related sesquiterpenes is governed by the stereochemical configuration of the bisabolyl cation intermediate, particularly at the C6 position (Jindal & Sunoj, 2012). Similar to the reactions catalyzed by most other enzymes in the TPS‐a2 clade II, the catalytic activity of ZmTPS9 likely proceeds through the hub of the complex carbocation web, the bisabolyl cation (C6–C1 closure). This carbocation then undergoes a cascade of rearrangements, hydride shifts, and cyclizations to form multiple sesquiterpene products (Figure S2). Of these, α‐santalene is derived by internal C2–C7 ring closure of the bisabolyl cation, while β‐bisabolene is formed by the deprotonation of the bisabolyl cation (Figure S2) (Hong & Tantillo, 2014; Jindal & Sunoj, 2012). Collectively, these findings demonstrate that ZmTPS9 functions as a multiproduct sesquiterpene synthase primarily producing α‐santalene and β‐bisabolene.

### Identification of a key residue of ZmTPS9

An amino acid sequence alignment of ZmTPS9 sequences from 18 different inbred lines, including B73 and W22, revealed high overall sequence identity (Figure S3). Among them, three of four α‐santalenoic acid‐deficient lines (B73, Oh43, and Mo18W) exhibited a deletion of one amino acid (Thr) at position 413 (Figure S3). To assess whether this deletion had functional consequences, we generated the mutant ZmTPS9‐W22‐ΔT413 through site‐directed mutagenesis and tested its enzymatic activity in *E. coli* using microbial co‐expression assays. Our results showed that the deletion of Thr413 completely abolished ZmTPS9‐W22 enzyme activity, indicating that this residue may play an important role in maintaining ZmTPS9 activity (Figure 4A).

To further investigate the structural impact of this deletion, we compared three‐dimensional models of ZmTPS9‐W22 and ZmTPS9‐B73 proteins generated through homology modeling using conserved terpene synthase motifs (Figure S4). Both variants retained hallmark features of terpene synthases, including the conserved DDxxD, NSE/DTE, and RxR motifs (Figure S4). Consistent with TPS‐a subfamily enzymes, ZmTPS9 adopts a characteristic two‐domain α‐helical fold comprising N‐terminal and C‐terminal catalytic regions (Christianson, 2017). Structural alignment revealed that the active site geometry and key FPP‐binding residues (Asp282, Asp286, Asn419, and Glu427) are conserved in both alleles, maintaining similar coordination environments (Figure S5). However, the ZmTPS9‐B73 model exhibited an inward shift of helix H2 within the C‐terminal domain (Figure 4B; Figure S6). Although Thr413 is located outside the active site, its absence in the B73 variant may alter local helical packing, thereby affecting overall protein folding and/or dynamic stability, which could contribute to the observed loss of catalytic activity in ZmTPS9‐B73. However, additional molecular dynamics studies and/or protein stability assays will be required to validate this hypothesis.

### ZmCYP71Z P450s catalyze α‐santalenoic acid biosynthesis

In typical plant sesquiterpenoid pathways, P450 enzymes catalyze sequential oxidation reactions that convert TPS products into more oxidized forms, such as carboxylic acids (Weitzel & Simonsen, 2013). Our previous studies identified three maize CYP71Z P450s (ZmCYP71Z16, ZmCYP71Z18 and ZmCYP71Z19) as key enzymes involved in the biosynthesis of multiple terpenoid defense metabolites (Ding et al., 2019, 2020). Among them, ZmCYP71Z19 functions exclusively within the sesquiterpenoid pathway, whereas ZmCYP71Z16 and ZmCYP71Z18 participate in both sesquiterpenoid and diterpenoid metabolism (Figure S7). In this study, we found that *ZmTPS9* and three *ZmCYP71Z* genes exhibited similar expression profiles in W22 stems following fungal elicitation (Figure S8), suggesting a potential functional connection in maize defense metabolism.

**Figure 5:**
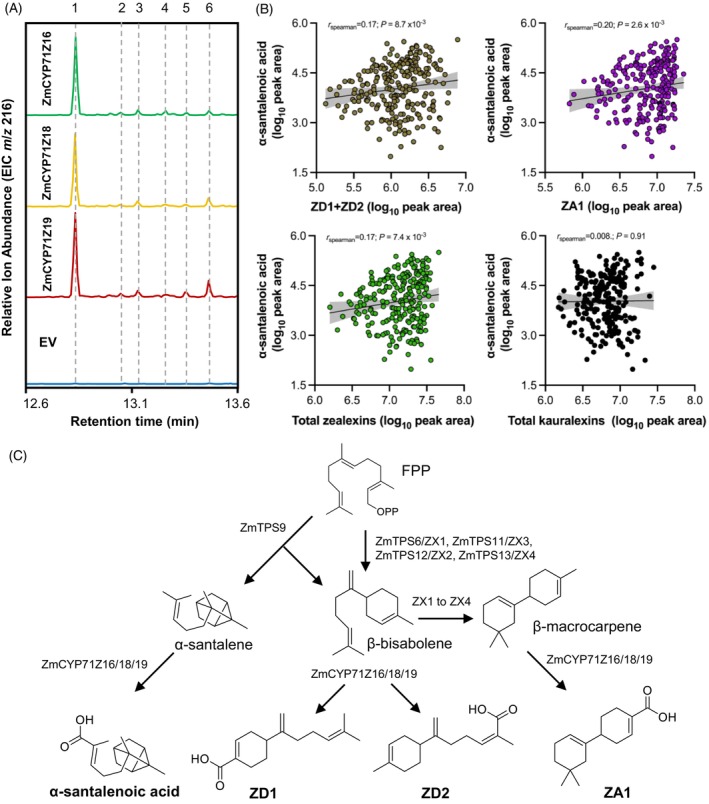
ZmTPS9 and ZmCYP71Z P450s collaboratively contribute to α‐santalenoic acid and zealexin biosynthesis. (A) GC‐MS analysis of transiently transformed *N. benthamiana* leaves co‐expressing *ZmTPS9* with individual *ZmCYP71Z* P450s genes (*ZmCYP71Z16*, *ZmCYP71Z18*, and *ZmCYP71Z19*). Co‐expression of *ZmTPS9* with the individual P450 genes resulted in the production of α‐santalenoic acid (analyte 1), ZD1 (analyte 4), and ZD2 (analyte 5), along with several additional unknown sesquiterpene acids (analytes 2, 3, and 6; See EI mass spectra in Figure S10). (B) α‐Santalenoic acid levels positively correlate with zealexin accumulation. Spearman correlation analysis was performed between α‐santalenoic acid and zealexin D1 (ZD1) + zealexin D2 (ZD2), ZA1, total zealexins, and total kauralexins across maize lines from the Goodman diversity panel (*n* = 237). Log₁₀‐transformed peak area values were used for the analysis. (C) Proposed model illustrating the contribution of ZmTPS9‐derived major sesquiterpene products to α‐santalenoic acid and distinct zealexin branches through oxidation by ZmCYP71Z P450s.

To evaluate whether these P450s are involved in α‐santalenoic acid biosynthesis, we performed *Agrobacterium*‐mediated transient expression assays in *Nicotiana benthamiana*. Co‐expression of *ZmTPS9*‐*W22* with each individual *ZmCYP71Z* P450 gene led to the consumption of sesquiterpene olefins and the formation of α‐santalenoic acid, ZD1, and ZD2, as well as several unidentified oxidized sesquiterpenoids (Figure 5A; Figures S9 and S10). These results support a role for the three ZmCYP71Z enzymes in α‐santalenoic acid biosynthesis in maize.

### ZmTPS9 contributes to the maize zealexin defense network

When *ZmTPS9‐W22* was co‐expressed with all three maize *CYP71Z* genes (*ZmCYP71Z16*, *ZmCYP71Z18*, and *ZmCYP71Z19*), both ZD1 and ZD2 were produced (Figure 5A), indicating that β‐bisabolene produced by ZmTPS9 serves as a precursor in zealexin biosynthesis. Supporting this, α‐santalenoic acid levels were positively correlated with ZD1 and ZD2 accumulation across maize lines from the Goodman diversity panel in response to fungal elicitation (Figure 5B), supporting a potential role for ZmTPS9 in the maize zealexin defense network.

β‐Bisabolene was reported to serve as an intermediate in the biosynthesis of β‐macrocarpene, as demonstrated by maize terpene synthases TPS6 and TPS11 (Köllner et al., 2008). This observation suggests that β‐bisabolene produced by ZmTPS9 may additionally contribute to the biosynthesis of β‐macrocarpene and its downstream zealexins. Consistent with this, we observed reduced zealexin levels in maize lines deficient in α‐santalenoic acid, whereas no correlation was found between α‐santalenoic acid and kauralexin accumulation (Figure 5B). These findings suggest that ZmTPS9‐derived β‐bisabolene may contribute to multiple zealexin biosynthetic branches. Additionally, we observed that co‐expression of *ZmTPS9‐W22* with each individual ZmCYP71Z P450 gene resulted in the formation of several unknown sesquiterpenoids (Figure 5A; Figure S10). Taken together, our results indicate that ZmTPS9, together with three ZmCYP71Z P450s, may contribute to multiple branches of the maize sesquiterpenoid defense network (Figure 5C).

## DISCUSSION

Species‐specific terpenoids play critical roles in plant chemical defense (Ding et al., 2021; Murphy & Zerbe, 2020). Across evolutionary time, plants from different lineages have independently evolved a rich diversity of terpenoid compounds in response to biotic and abiotic pressures (Pichersky & Raguso, 2018; Smit & Lichman, 2022). In this study, we identified α‐santalenoic acid, a tricyclic sesquiterpenoid derivative, as a pathogen‐inducible antibiotic in maize (Figure 1). α‐Santalenoic acid exhibited potent antifungal activity against two major maize pathogens, *F. graminearum* and *F. verticillioides* (Figure 1C), highlighting its potential function in maize innate immunity. Similar terpenoid derivatives have been identified in other plant species such as *Solanum habrochaites*, where they are major constituents of glandular trichome secretions and contribute to resistance against insect herbivory (Frelichowski & Juvik, 2001; Therezan et al., 2021). The conversion of sesquiterpene hydrocarbons into polar, bioactive compounds like acids and aldehydes in response to pathogen attack is a common strategy observed in many plant species. For instance, in maize, β‐bisabolene and β‐macrocarpene are oxidized to zealexins, a class of antimicrobial acidic sesquiterpenoids (Ding et al., 2020; Huffaker et al., 2011). In cotton, δ‐cadinene is modified into gossypol and related defensive sesquiterpenes (Chen et al., 1995; Tian et al., 2019). Given the insecticidal and antibacterial activities of α‐santalenoic acid (Coates et al., 1988, Frelichowski & Juvik, 2001; Xu et al., 2014) (Figure 1C), the identification of its biosynthetic genes in maize provides valuable insights into lineage‐specific, terpenoid‐mediated defense mechanisms.

Although α‐santalene synthases have been described in sandalwood (Jones et al., 2011) and tomato (Therezan et al., 2021; Wang et al., 2020), homologous enzymes were not initially apparent in maize. Through a combination of mGWAS, linkage mapping, and enzyme analyses, we identified ZmTPS9 as the key enzyme responsible for α‐santalene biosynthesis (Figure 2). Phylogenetic analysis placed ZmTPS9 within the TPS‐a2 clade II (Figure 3A), a subfamily largely composed of sesquiterpene synthases in angiosperms (Luck et al., 2020; Zhou & Pichersky, 2020). Notably, ZmTPS9 is part of a 921‐kb genomic region on chromosome 10 that includes *ZmTPS4*, *ZmTPS5*, and *ZmTPS10*, known to generate overlapping sesquiterpene products (Köllner et al., 2004; Schnee et al., 2006). The proximity and sequence similarity among these genes suggest recent duplication events, contributing to functional diversification and expansion of terpenoid metabolic capacity (Li et al., 2021; Luck et al., 2020). Within this region, we also identified nonfunctional *ZmTPS9* alleles in α‐santalenoic acid‐deficient lines (Figure 2A), consistent with previous findings of gene loss or pseudogenization in other maize TPS loci (Ding et al., 2020; Köllner et al., 2004; Richter et al., 2016).

Functionally, ZmTPS9 is a multiproduct sesquiterpene synthase that primarily produces α‐santalene and β‐bisabolene (Figure 3B), the latter being a known precursor to β‐macrocarpene (Köllner et al., 2008). In addition to these dominant products, ZmTPS9 also produces several minor sesquiterpenes with varying relative abundances (Figure 3B; Table S1), reflecting its broad substrate flexibility and catalytic promiscuity. It is noteworthy that many maize TPSs exhibit broad catalytic promiscuity, often producing overlapping sets of sesquiterpene scaffolds (Köllner et al., 2006; Luck et al., 2020). However, the biological relevance of such catalytic activities depends strongly on context‐specific gene expression. Several TPSs may encode enzymes capable of forming particular sesquiterpene products *in vitro*, yet remain transcriptionally silent under specific physiological or environmental conditions (Ding et al., 2020). Integrating metabolite‐based genetic mapping with expression profiling therefore provides an effective strategy for linking biosynthetic genes, including not only TPSs but also downstream tailoring enzymes, to physiologically relevant terpenoid defense pathways (Wu et al., 2023).

The metabolic versatility of ZmTPS9 suggests that it functions as an important contributor to maize terpenoid defenses by providing multiple intermediates for downstream oxidation. A positive correlation between α‐santalenoic acid and various zealexin branches (Figure 5B) further supports the involvement of ZmTPS9 in multiple arms of the maize sesquiterpenoid defense network. Similar catalytic diversity has been reported for other sesquiterpene synthases, including SaSSy from *S. album* (Jones et al., 2011), CmCJTPS3 from *C. morifolium* (Zhang et al., 2022), and SsT2 from *S. habrochaites*, all of which divert bisabolyl cation intermediates into structurally diverse terpenes (Hong & Tantillo, 2014; Jindal & Sunoj, 2012; Therezan et al., 2021). This catalytic plasticity reflects a broader evolutionary trend where plants leverage TPS‐mediated electrophilic reaction cascades to generate diverse metabolites from a single substrate (Hong & Tantillo, 2014; Vattekkatte et al., 2018).

ZmTPS9's ability to form multiple products is governed by its active‐site architecture, which controls reaction outcomes through subtle structural features. Similar to maize TPS4, which utilizes dual active‐site pockets to direct different cyclization reactions (Köllner et al., 2006), ZmTPS9 may rely on subtle differences in active‐site architecture or amino acid positioning to influence product outcomes, although further structural or mutational analyses would be required to confirm this. In this study, we identified a threonine residue at position 413 as critical for enzymatic activity. Deletion of Thr413, a residue adjacent to the conserved NSE/DTE motif, led to the predicted distortion of helix H2 and complete loss of catalytic function (Figure 4). Analogous structural effects have been reported for other TPSs, including MtTPS5 from *Medicago truncatula* and (+)‐limonene synthases in *Aquilegia* (Kampranis et al., 2007; Vattekkatte et al., 2018; Yang et al., 2022). Such findings underscore the importance of peripheral residues in maintaining the integrity and dynamics of TPS catalytic pockets (Greenhagen et al., 2006).

Downstream of sesquiterpene scaffold formation, functional modifications are often catalyzed by P450s, resulting in various bioactive compounds (Banerjee & Hamberger, 2018). In this study, we found that the maize P450s, ZmCYP71Z16, ZmCYP71Z18, and ZmCYP71Z19, oxidize ZmTPS9‐derived hydrocarbons to form α‐santalenoic acid and zealexins (Figure 5). Similar functional diversification has been reported in sandalwood, where SaCYP76F and SaCYP736A enzymes selectively oxidize santalene derivatives to yield α‐santalol and related products (Celedon et al., 2016; Diaz‐Chavez et al., 2013). Notably, both CYP76 and CYP71Z families belong to the CYP71 clan, which often oxidizes terpenes (Hansen et al., 2021). Similar sesquiterpenoid scaffolds and oxidized derivatives, such as α‐santalene and α‐santalenoic acid, are also produced in distantly related plant species including *Santalum album* (sandalwood) and *Solanum habrochaites* (wild tomato) (Gonzales‐Vigil et al., 2012; Jones et al., 2011). In these species, α‐santalene is synthesized by terpene synthases from different TPS subfamilies than ZmTPS9, supporting the independent evolution of this catalytic function. The repeated emergence of santalene‐type metabolites in diverse lineages exemplifies convergent evolution in plant specialized metabolism, likely driven by shared ecological pressures.

Together, our findings demonstrate how ZmTPS9 and ZmCYP71Z P450s collaboratively orchestrate a responsive sesquiterpenoid defense network in maize. The catalytic versatility of ZmTPS9 enables the generation of diverse terpene scaffolds, while the substrate flexibility of ZmCYP71Z enzymes facilitates the biosynthesis of numerous downstream defense metabolites. Moreover, these ZmCYP71Z enzymes also play a pivotal role in the broader metabolic network, contributing to both sesquiterpenoid and diterpenoid pathways that yield key phytoalexins, such as kauralexins, dolabralexins, and zealexins (Ding et al., 2021). The dynamic modular interactions between TPSs and P450s highlight an evolutionary strategy in which substrate plasticity and enzyme diversification underpin metabolic adaptability. This coordinated system not only reinforces maize immune resilience but also provides a valuable foundation for biotechnological applications aimed at enhancing crop protection.

## MATERIALS AND METHODS

### Plant and fungal materials

Maize seeds from the Goodman diversity panel for metabolite‐based mapping and the Nested Association Mapping (NAM) RILs (B73 × M162W) for linkage analysis were provided by G. Jander (Boyce Thompson Institute) and P. Balint‐Kurti (US Department of Agriculture, Agricultural Research Service [USDA‐ARS]) (Flint‐Garcia et al., 2005). Maize seeds were germinated in Metro‐Mix 200 (Sun Gro Horticulture Distribution) supplemented with 14‐14‐14 Osmocote (Scotts Miracle‐Gro) and grown in a greenhouse as previously described (Ding et al., 2017). To avoid differences in plant response due to fungal expression and action, heat‐killed *F. venenatum* (strain PTA‐2684) hyphae were commercially obtained (Monde Nissin Corporation Co., Santa Rosa, Laguna, Philippines) and used as a non‐infectious elicitor in all the mapping experiments (Ding et al., 2019, 2020). Fungus‐treated maize stems were harvested after 5 days, frozen in liquid nitrogen, ground to a fine powder, and stored at −80°C for further metabolite analyses.

Fungal cultures of *F. graminearum* (NRRL stock no. 31084) and *F. verticillioides* (NRRL stock no. 20956) were grown on V8 agar for 12 days before the spores were quantified and used (Huffaker et al., 2011).

### Maize stem challenge with heat‐killed *Fusarium*

Thirty‐five‐day‐old maize plants were slit in the center using a scalpel across both sides of the stem to create a 10‐cm longitudinal incision. This incision wounded the upper nodes, internodes, and the basal sections of unexpanded leaves. Approximately 500 μl of commercially prepared heat‐killed *F. venenatum* hyphae was applied to each slit stem, and the incision site was then sealed with clear plastic packing tape to minimize tissue desiccation. The Goodman diversity panel and the B73 × M162W RIL subpopulation were planted in summer 2016 at the Biology Field Station, UCSD (Table S2) (Ding et al., 2019).

Maize stems were harvested 5 days after elicitation, flash‐frozen in liquid nitrogen, ground into a fine powder, and stored at −80°C for subsequent metabolite analysis.

### Analyses and identification of metabolites

Maize and *N. benthamiana* tissue samples were flash‐frozen in liquid nitrogen, ground to a fine powder, and stored at −80°C until analysis. Fifty milligrams of tissue aliquots were extracted with 300 μl of H_2_O:1‐propanol:HCl (1:2:0.005) and homogenized, followed by the addition of 1 ml MeCl_2_. The mixture was then re‐homogenized and centrifuged, yielding a top aqueous layer and a lower MeCl_2_:1‐propanol layer. The lower MeCl_2_:1‐propanol layer was transferred to a 4 ml glass vial, derivatized with trimethylsilyldiazomethane, and processed by vapor‐phase extraction as previously described (Schmelz et al., 2004, 2011). GC‐MS analysis of extracts was performed on an Agilent (Santa Clara, CA, USA) 6890 GC coupled to an Agilent 5973 (EI) mass selective detector (interface temperature, 250°C; mass temperature, 150°C; source temperature, 230°C; electron energy, 70 eV) with a DB‐35MS column (Agilent; 30 m × 250 μm × 0.25 μm film). The sample was injected by pulsed splitless injection with an initial oven temperature of 45°C. The temperature was maintained at 45°C for 2.25 min, ramped to 300°C at 20°C min^−1^, and held at 300°C for 5 min. The mass spectrometer interface temperature was set at 250°C. The mass spectrometer operated at 70 eV of electron energy; quadrupole and ion‐source temperatures were 150°C and 230°C, respectively. Products were identified through comparison of the GC‐MS retention times and mass spectra with those from standards and enzymatically produced authentic compounds. *Escherichia coli*‐expressed enzyme products were subjected to GC‐MS analysis. MS data from a mass‐to‐charge ratio (*m*/*z*) range of 90–600 were collected after a 9‐min solvent delay.

GC‐MS analysis of products from the ZmTPS9 *in vitro* assay was conducted using an Agilent 7890B gas chromatograph coupled to a 5977 Extractor XL mass spectrometer (70 eV) with a helium carrier gas flow rate of 1.2 ml min^−1^. Samples (1 μl) were injected in pulsed splitless mode at 250°C onto an HP5‐MS column (30 m length, 250 μm internal diameter, 0.25 μm film thickness). The oven temperature was initially set to 50°C (3 min hold), ramped at 20°C min^−1^ to 300°C, and held for 3 min. Mass spectra were recorded over an *m*/*z* range of 90–600 following a 9‐min solvent delay. Opoponax oil and essential oils from *Aloysia sellowii* (hydrocarbon fraction) and *Phoebe porosa* were used as reference standards for the identification of ZmTPS9 products (Figure S11). Specifically, 7‐*epi*‐sesquithujene, *cis*‐α‐bergamotene, *trans*‐α‐bergamotene, *ar*‐curcumene, β‐bisabolene, and sesquisabinene A were identified using *Phoebe porosa* essential oil, while sesquisabinene B and sesquithujene were identified using *Aloysia sellowii* oil. α‐Santalene was confirmed using Opoponax oil. Additionally, other ZmTPS9‐derived products were identified through comparison with reference mass spectra from the NIST and Wiley spectral databases. Essential oils used for TPS product identification were kindly provided by Wilfried A, König, Hamburg, Germany.

### Genetic mapping of fungal‐elicited defense

Metabolite‐based GWAS was carried out using the Unified Mixed Linear Model in TASSEL 5.0 and the GAPIT R package (Ding et al., 2017; Lipka et al., 2012; Zhang et al., 2010). SNPs with less than 20% missing genotype data and minor allele frequencies greater than 5% from both an Illumina 50 K array and a genotyping‐by‐sequencing strategy (GBS) were employed in the association analysis. To improve the association analysis, the kinship matrix (K) and the population structure (Q) library were constructed using the SMARTer RACE 5′/3′ Kit (Clontech, Mountain View, CA, USA), as previously described (Ding et al., 2017). Linkage analysis was performed on α‐santalenoic acid production in the B73 × M162W NAM recombinant line subpopulation. QTL analysis was conducted using composite interval mapping implemented in Windows QTL Cartographer v.2.5 software (https://brcwebportal.cos.ncsu.edu/qtlcart/WQTLCart.htm), as previously described (Ding et al., 2020; Wen et al., 2014). The genetic marker data for the RILs were downloaded from www.panzea.org.

### Heterologous production of ZmTPS9 products in *E. coli*

The construction of cDNA RACE libraries from maize lines B73 and W22 was performed using established methods as previously described (Ding et al., 2019). Briefly, total RNA was extracted from 35‐day‐old B73 and W22 meristem tissues elicited with heat‐killed *F. venenatum* hyphae for 48 h using a NucleoSpin® RNA Plant Kit (Takara Bio, San Jose, CA, USA) according to the manufacturer's protocol. Approximately 2 μg of total RNA was treated with TURBO DNA‐free™ (Ambion, Carlsbad, CA, USA) to remove genomic DNA contamination and subsequently used for constructing a 5′ rapid amplification of cDNA ends (5'RACE) library using the SMARTer™ RACE 5′/3′ Kit (Clontech). The full‐length open reading frames (ORFs) of *ZmTPS9‐W22* and *ZmTPS9‐B73* were amplified by specific primers (Table S3) and cloned into the pET‐Duet1 expression vector (EMD Millipore, Burlington, MA, USA), resulting in the constructs pET‐Duet1:ZmTPS9‐B73 and ZmTPS9‐W22. The full‐length gene of maize *farnesyl diphosphate synthase 3* (*ZmFPS3*, *Zm0001d043727*) was inserted into the pCOLA‐Duet1 expression vector (EMD Millipore).

Individual TPS constructs were then co‐expressed with pCOLADuet1:ZmFPS3 in BL21DE3‐C41 *E. coli* cells (Lucigen, Middleton, WI, USA). The expression of pCOLA‐Duet1:ZmFPPS3 only was used as a control. Transformed *E. coli* cells were cultured at 37°C in 50 ml Terrific Broth (TB) medium with the appropriate antibiotics until an OD_600_ of approximately 0.6 was reached. The temperature was then reduced to 16°C, and 1 mm isopropyl 1‐thio‐β‐d‐galactopyranoside (IPTG) was added. Enzyme products were extracted using a 1:1 mixture of ethyl acetate and hexane (v/v) and concentrated under N_2_ stream. Sesquiterpene volatiles were collected by N_2_ gas over the solvent extracts at a flow rate of 600 ml min^−1^ and trapped the volatiles on inert filters packed with 50 mg of HayeSep Q polymer adsorbent (80–100 μm mesh; Sigma‐Aldrich, Saint Louis, MO, USA). Trapped compounds were eluted with 150 μl of methylene chloride and analyzed by GC‐MS.

### *In vitro* assay for ZmTPS9 activity

The complete open reading frame of ZmTPS9‐W22 was cloned into the vector pET100/D‐TOPO (Invitrogen, Carlsbad, CA, USA). For heterologous expression in *E. coli*, the plasmid was transformed into the strain BL21 Codon Plus (Invitrogen). Protein expression was induced by adding isopropyl β‐d‐1‐thiogalactopyranoside (IPTG) to a final concentration of 1 mm. Cells were harvested by centrifugation at 4000 ***g*** for 6 min and lysed by four 30‐sec pulses of sonication in chilled extraction buffer (50 mm MOPS, pH 7.0, 5 mm MgCl_2_, 5 mm sodium ascorbate, 0.5 mm phenylmethylsulfonyl fluoride, 5 mm DTT, and 10% [v/v] glycerol). Following lysis, cellular debris was removed by centrifugation at 14 000 ***g***, and the supernatant was desalted into assay buffer (10 mm MOPS, pH 7.0, 1 mm DTT, and 10% [v/v] glycerol) using an Econopac 10DG desalting column (Bio‐Rad, Hercules, CA, USA). Enzyme assays were carried out in 1‐ml Teflon‐lined, screw‐cap GC vials containing 50 μl of the clarified protein extract and 50 μl of assay buffer supplemented with 10 μm (*E*,*E*)‐farnesyl diphosphate (FPP) and 10 mm MgCl_2_. Volatile terpene products were captured using solid‐phase microextraction (SPME) fibers and analyzed by gas chromatography–mass spectrometry (GC‐MS), as described in the “Analyses and identification of metabolites” section.

### Site‐directed mutagenesis

The site‐directed mutation (ΔT413) of ZmTPS9‐W22 in the vector pET‐Duet1 was introduced by whole‐plasmid PCR amplification with site‐specific sense and antisense oligonucleotides (Table S4). The PCR product was then incubated with *Dpn*I at 37°C for 1 h to digest the parental double‐stranded DNA (methylated) before transformation into *E. coli* (BL21DE3‐C41). The presence of the expected mutation in the resulting construct was identified by DNA sequencing.

### Transient co‐expression assays in *N. benthamiana*

*ZmTPS9‐W22* was subcloned into the vector pLIFE33 using methods described previously (Table S5) (Ding et al., 2020). The vectors pLIFE33‐ZmCYP71Z16, pLIFE33‐ZmCYP71Z18, and pLIFE33‐ZmCYP71Z19 were derived from prior work (Ding et al., 2020). For transient expression in *N. benthamiana*, pLIFE33 vectors containing individual targeted genes and pEarleyGate100 with ElHMGR^159–582^(Sadre et al., 2019) were electroporated into *Agrobacterium tumefaciens* strain GV3101. *Agrobacterium* cultures were grown at 28°C for 24 h in Luria–Bertani (LB) medium containing 50 mg L^−1^ kanamycin, 30 mg L^−1^ gentamicin, and 50 mg L^−1^ rifampicin. The resulting cells were collected by centrifugation at 3800 ***g*** for 10 min, washed, and resuspended in 10 mm MES buffer (pH 5.6) with 10 mm MgCl_2_ to an optical density at 600 nm (OD_600_) of 0.8. The selected bacterial suspensions were mixed together in equal proportions and then syringe‐infiltrated into the fully expanded leaves of 6‐week‐old *N. benthamiana* plants (Bach et al., 2014). In addition, an *Agrobacterium* strain harboring a plasmid encoding the anti‐posttranscriptional gene silencing protein p19 was added in equal amounts to each combination to ensure high levels of transient expression (Voinnet et al., 2003). Three days post inoculation, inoculated leaves were frozen in liquid N_2_, ground to a powder, and stored at −80°C until further GC‐MS analyses.

### *In vitro* bioassays of α‐santalenoic acid activity as an antifungal agent

*In vitro* antifungal assays of α‐santalenoic acid (CAS: 74642‐79‐8; Sigma‐Aldrich) were analyzed using the Clinical and Laboratory Standards Institute M38‐A2 guidelines as detailed previously (Schmelz et al., 2011). Briefly, fungal growth at 30°C in broth medium was monitored using a Synergy4 (BioTech Instruments, Winooski, VT, USA) reader with a 96‐well microtiter plate‐based method through periodic measurements of changes in the OD_600_. Each well contained 200 μl of initial fungal inoculum (2.5 × 10^4^ conidia ml^−1^) with 0.5 μl of either pure dimethyl sulfoxide (DMSO) or DMSO containing α‐santalenoic acid.

### Multiple sequence alignment and phylogenetic analysis

Terpene synthase genes from six Poaceae species (*Oryza sativa*, *Sorghum bicolor*, *Zea mays*, *Panicum virgatum*, *Setaria italica*, *Brachypodium distachyon*), as well as from *Arabidopsis thaliana* were analyzed (Table S6). The sequences were downloaded from the NCBI database (https://www.ncbi.nlm.nih.gov/) and the Phytozome database (https://phytozome‐next.jgi.doe.gov/). All sequences were aligned using MAFFT (v7.505, −auto option), and gap sequences were trimmed using TrimAl (Capella‐Gutiérrez et al., 2009). The maximum likelihood tree was constructed with IQ‐Tree (v2.1.2; with best‐fit models and 1000 bootstrapped replicates). TPS subfamilies (TPS‐a, TPS‐g, TPS‐b, TPS‐c, and TPS‐e/f clades) are categorized according to Luck et al. (2020).

### Homology modeling of a 3D structure for ZmTPS9

The homology model of ZmTPS9 was built based on sequence alignment using multi‐template modeling. The following templates were selected: (−)‐drimenol synthase from *Persicaria hydropiper* (PDB ID, 7CJY), (+)‐delta‐cadinene synthase from *Gossypium arboretum* (PDB ID, 3G4D), santalene synthase from *S. album* (PDB ID, 5ZZJ), sesquisabinene B synthase from *S. album* (PDB ID, 6A1D), 5‐*epi*‐aristolochene synthase from *N. tabacum* (PDB ID, 5EAT) and 5‐*epi*‐aristolochene synthase from *N. tabacum* (PDB ID, 5IK0). The Modeler v10.3 program (https://salilab.org/modeller/) was used to create 500 models, and the models were sorted according to their DOPE score, where models with the lowest DOPE score were selected for additional optimization (Webb & Sali, 2014). The AMBER14 force field was used, and the protein model was initially cleaned and optimized (Maier et al., 2015). Structure superposition was performed to superimpose the substrate FPP in the crystal structure 5IKO, followed by molecular mechanics optimization (Koo et al., 2016). Finally, PROCHECK and VERIFY 3D were used to evaluate the models. The Ramachandran plot showed that 92.4% and 93.7% of the amino acid residues in the ZmTPS9‐W22 and ZmTPS9‐B73 protein models were located in the core region, and only 0.4% were located in the forbidden zone of the twist angle (Figure S12). Overall, 99.6% of the amino acids were within the reasonable range and conformed to the stereochemical energy rule (Figure S12).

### Statistical analyses

Statistical analyses were conducted using GraphPad Prism 8.0 (GraphPad Software, Inc., Boston, MA, USA) and JMP Pro 13.0 (SAS Institute Inc., Cary, NC, USA). One‐way analyses of variance (anova) were performed to evaluate significant differences. Tukey tests were used to correct for multiple comparisons between the control and treatment groups. Student's unpaired *t*‐tests were used for pairwise comparisons. *P* values <0.05 were considered to indicate statistical significance. Additionally, Spearman correlations and *P*‐values were also calculated between α‐santalenoic acid and different branches of zealexins and total kauralexin levels (Table S7).

## AUTHOR CONTRIBUTIONS

YD, TGK, AH, and EAS conceived and designed the experiments. YD, MW, and TGK performed the mass spectrometry (MS) experiments and analyzed MS‐based metabolite data. YD conducted genetic mapping, gene cloning, site‐directed mutagenesis, and enzyme co‐expression assays. ZS and SPB analyzed ZmTPS9 protein levels. TGK and YD carried out the identification of ZmTPS9 sesquiterpene products. MW and EP performed ZmTPS9 structural modeling. MW and YD wrote the manuscript with input from all authors.

## CONFLICT OF INTEREST

The authors have not declared a conflict of interest.

## Supporting information


**Figure S1.** Induction of ZmTPS9 transcript and protein levels in W22 stems upon elicitation with heat‐killed *Fusarium venenatum*.
**Figure S2.** A mechanistic overview of the biosynthesis of 15 distinct sesquiterpenes catalyzed by ZmTPS9.
**Figure S3.** ZmTPS9 amino acid sequence comparison across selected maize inbred lines.
**Figure S4.** Three‐dimensional model of the maize terpene synthase ZmTPS9.
**Figure S5.** Substrate‐binding pocket and FPP docking in ZmTPS9.
**Figure S6.** Amino acid sequence comparison of ZmTPS9 and other plant TPSs.
**Figure S7.** Functional overview of three ZmCYP71Z P450 enzymes in maize terpenoid metabolism.
**Figure S8.** Heat map depicting temporal protein expression dynamics in W22 stem tissues following elicitation with heat‐killed *Fusarium venenatum*.
**Figure S9.** GC–MS analysis of transiently transformed *N. benthamiana* leaves co‐expressing *ZmTPS9* with individual *ZmCYP71Z* P450 genes (*ZmCYP71Z16*, *ZmCYP71Z18*, *and ZmCYP71Z19*) or with the empty vector (EV) control.
**Figure S10.** EI mass spectra of unidentified sesquiterpene acids detected in *N. benthamiana* leaf tissues transiently co‐expressing *ZmTPS9* with individual P450 genes (*ZmCYP71Z16*, *ZmCYP71Z18*, *or ZmCYP71Z19*), as well as with the empty vector (EV) control.
**Figure S11.** Identification of ZmTPS9 sesquiterpene products. Opoponax oil and essential oils from *Aloysia sellowii* (hydrocarbon fraction) and *Phoebe porosa* were used as reference standards for the identification of ZmTPS9 products.
**Figure S12.** Ramachandran plots and verify3D scores of TPS9 models.

**Table S1.** Sesquiterpene olefin products generated by ZmTPS9‐W22 and their relative abundances.
**Table S2.** Maize mapping lines used for metabolite‐based genome wide association studies (mGWAS) in the Goodman diversity panel and Quantitative Trait Loci (QTL) mapping in the Nested Association Mapping (NAM) subpopulation B73 × M162W.
**Table S3.** Gene sequences used in enzyme co‐expression studies including native sequences, synthetic sequences, and codon‐optimized sequences for expression in *E. coli* or in *N. benthamiana*.
**Table S4.** Primer sequences for site‐directed mutagenesis for generate ZmTPS9 mutants.
**Table S5.** Primers used for gene cloning into the pLIFE33 expression vector from cDNA.
**Table S6.** Terpene synthase genes from six Poaceae species and *Arabidopsis thaliana* for phylogenetic analyses in this study.
**Table S7.** Metabolite levels, including zealexins, kauralexins, and α‐santalenoic acid, measured as peak areas in fungal‐elicited stem tissues from maize lines in the Goodman Diversity Panel.

## Data Availability

All plant accessions used, gene identifiers, unique protein sequences, heterologously expressed genes, and web links to publicly available datasets are listed in detail in the “Materials and Methods” section and Tables S1–S7. All unique materials are readily available from the authors on request.
