# Supplementary material for: A multifunctional sesquiterpene synthase integrates with cytochrome P450s to reinforce the terpenoid defense network in maize
Source: Plant J. 2025 Nov 14;124(3):e70575. doi: 10.1111/tpj.70575 (PMC12617997; doi:10.1111/tpj.70575)
Supplement: Supplementary file 1 — Figure S1. Induction of ZmTPS9 transcript and protein levels in W22 stems upon elicitation with heat‐killed Fusarium venenatum. Figure S2. A mechanistic overview of the biosynthesis of 15 distinct sesquiterpenes catalyzed by ZmTPS9. Figure S3. ZmTPS9 amino acid sequence comparison across selected maize inbred lines. Figure S4. Three‐dimensional model of the maize terpene synthase ZmTPS9. Figure S5. Substrate‐binding pocket and FPP docking in ZmTPS9. Figure S6. Amino acid sequence comparison of ZmTPS9 and other plant TPSs. Figure S7. Functional overview of three ZmCYP71Z P450 enzymes in maize terpenoid metabolism. Figure S8. Heat map depicting temporal protein expression dynamics in W22 stem tissues following elicitation with heat‐killed Fusarium venenatum. Figure S9. GC–MS analysis of transiently transformed N. benthamiana leaves co‐expressing ZmTPS9 with individual ZmCYP71Z P450 genes (ZmCYP71Z16, ZmCYP71Z18, and ZmCYP71Z19) or with the empty vector (EV) control. Figure S10. EI mass spectra of unidentified sesquiterpene acids detected in N. benthamiana leaf tissues transiently co‐expressing ZmTPS9 with individual P450 genes (ZmCYP71Z16, ZmCYP71Z18, or ZmCYP71Z19), as well as with the empty vector (EV) control. Figure S11. Identification of ZmTPS9 sesquiterpene products. Opoponax oil and essential oils from Aloysia sellowii (hydrocarbon fraction) and Phoebe porosa were used as reference standards for the identification of ZmTPS9 products. Figure S12. Ramachandran plots and verify3D scores of TPS9 models. [file TPJ-124-0-s002.pdf]

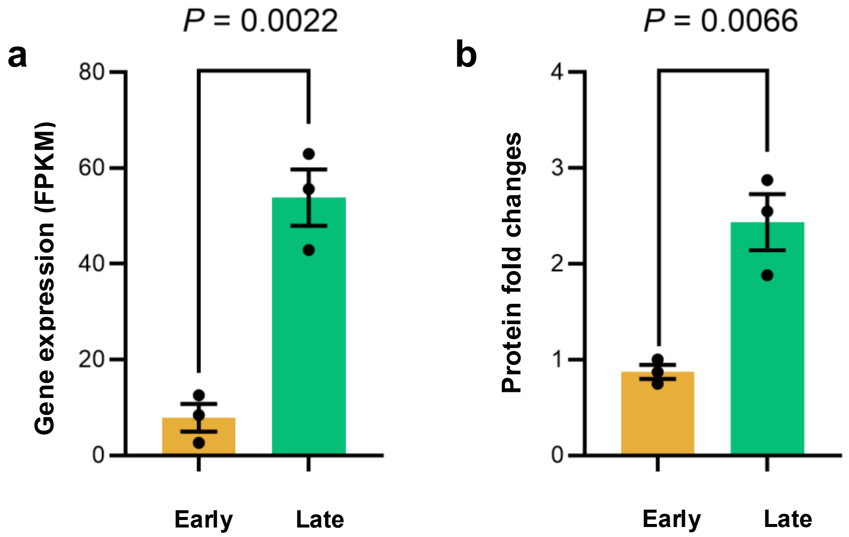

Figure S1. Induction of *ZmTPS9* transcript and protein levels in W22 stems upon elicitation with heat-killed *Fusarium venenatum.*
a, *ZmTPS9* transcript levels in W22 stem tissue treated with heat-killed *F. venenatum*, based on RNA-seq data from Supplementary Table 1 (Ding et al., 2020). Expression values are shown as fragments per kilobase of exon per million mapped reads (FPKM + 0.01; B73 RefGen V4) across a time-course following stem incision and elicitation. **b**, Protein fold changes of ZmTPS9 in W22 stems upon fungal elicitation, based on proteomics data from Supplementary Table 10 (Ding et al., 2020). Protein abundance was compared between Early (0, 2, and 4 hours) and Late (72, 96, and 120 hours) time points. Statistical analysis was performed using Student's *t*-test on three biologically independent replicates ($n = 3$). Asterisks indicate significant differences ($P < 0.05$).

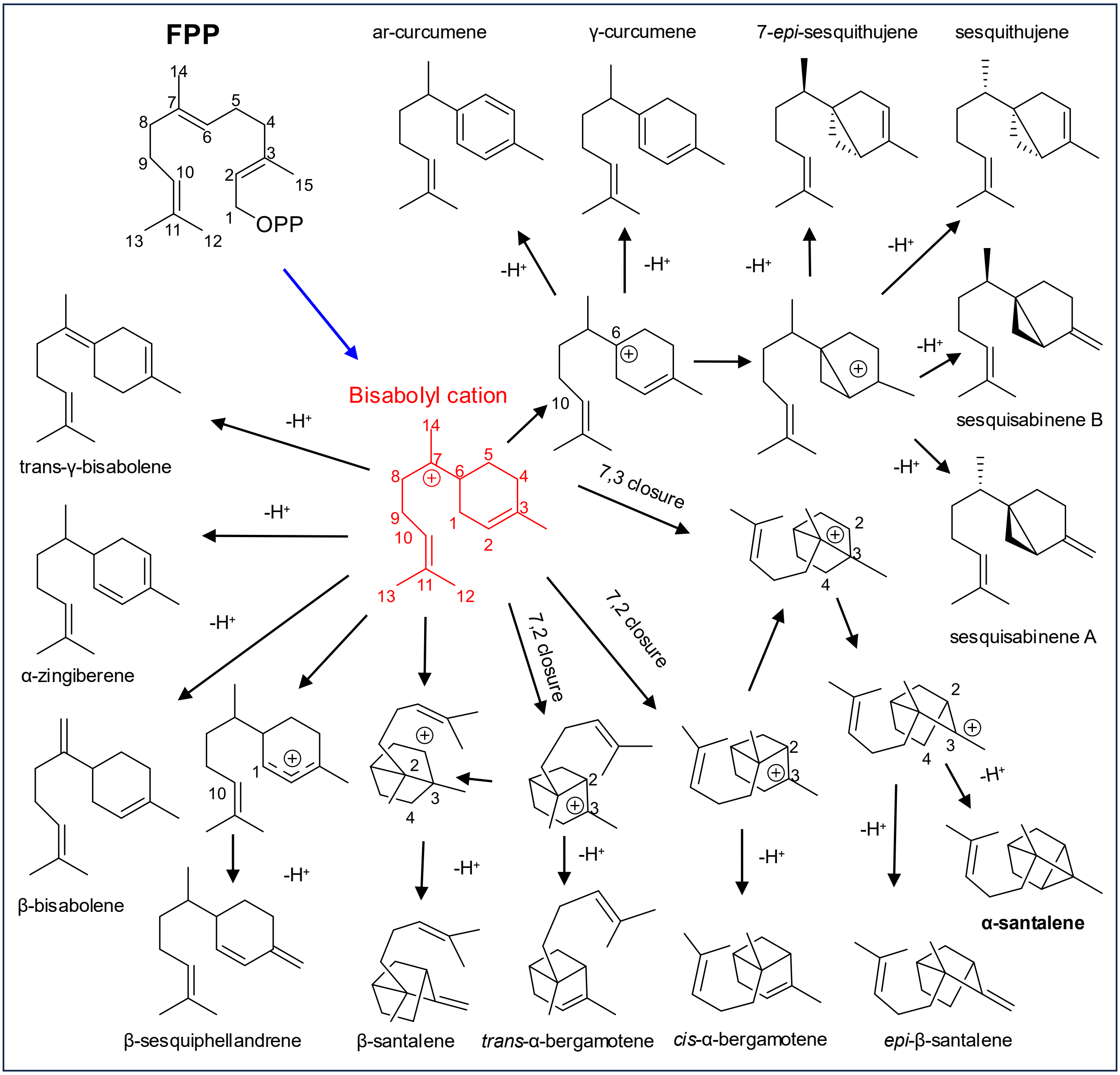

Figure S2. A mechanistic overview of the biosynthesis of 15 distinct sesquiterpenes catalyzed by ZmTPS9.

```
                   ....|....| ....|....| ....|....| ....|....| ....|....| ....|....| ....|....| ....|....| ....|....| ....|....| ....|....| ....|....|
                           5         15         25         35         45         55         65         75         85         95        105        115
Zm00004b038743_W22          MAPPPPAL-- -----AEETA AFHPSLWGDF FLTYQPPTAP QQACMKERAE VLREDVRKIL MGPCELPETL GLILTLQRLA LDYYYENEID KLLHCIY-DD DDSDYDDKDL NLVSLRFYLL
Zm00001d024477_B73          MAPPPPAL-- -----AEETA AFHPSLWGDF FLTYQPPTAP QQACMKERAE VLREDVRKIL MGPCELPETL GLILTLQRLA LDYYYENEID KLLHCIY-DD DDSDYDDKDL NLVSLRFYLL
Zm00039ab424100_Oh43        MAPPPPAL-- -----AEETA AFHPSLWGDF FLTYQPPTAP QQACMKERAE VLREDVRKIL MGPCELPETL GLILTLQRLA LDYYYENEID KLLHCIY-DD DDSDYDDKDL NLVSLRFYLL
Zm00022ab421380_CML228      MAPPPPALLA GSSKAAEETT SFHPSLWGDF FLTYQPPTAP QQACMKERAE VLREDVRKIL MGPCELPETL GLILTLQRLA LDYYYEKEID KLLHCIYNDD DDSDYDDKDL NLVSLRFYLL
Zm00023ab427130_CML247      MAPPPPAL-- -----AEETA AFHPSLWGDF FLTYQPPTAP QQACMKERAE VLREDVRKIL MGPCELPETL GLILTLQRLA LDYYYENEID KLLHCIY-DD DDSDYDDKDL NLVSLRFYLL
Zm00024ab424620_CML277      MAPPPPAL-- -----AEETA AFHPSLWGDF FLTYQPPTAP QQACMKERAE VLREDVRKIL MGPCELPETL GLILTLQRLA LDYYYENEID KLLHCIY-DD DDSDYDDKDL NLVSLRFYLL
Zm00026ab417460_CML333      MAPPPPALLA GSSKAAEETT SFHPSLWGDF FLTYQPPTAP QQACMKERAE VLREDVRKIL MGPCELPETL GLILTLQRLA LDYYYEKEID KLLHCIYNDD DDSDYDDKDL NLVSLRFYLL
Zm00019ab401530_CML52       MAPPPPALLA GSSKAAEETT SFHPSLWGDF FLTYQPPTAP QQACMKERAE VLREDVRKIL MGPCELPETL GLILTLQRLA LDYYYEKEID KLLHCIYNDD DDSDYDDKDL NLVSLRFYLL
Zm00020ab421560_CML69       MAPPPPALLA GSSKAAEETT SFHPSLWGDF FLTYQPPTAP QQACMKERAE VLREDVRKIL MGPCELPETL GLILTLQRLA LDYYYENEID KLLHCIY-DD DDSDYDDKDL NLVSLRFYLL
Zm00030ab419080_Ki11        MAPPPPALLA GSSKAAEETT SFHPSLWGDF FLTYQPPTAP QQACMKERAE VLREDVRKIL MGPCELPETL GLILTLQRLA LDYYYENEID KLLHCIY-ND DDSDYDDKDL SLVSLRFYLL
Zm00034ab441680_Mo18W       MAPPPPAL-- -----AEETA AFHPSLWGDF FLTYQPPTAP QQACMKERAE VLREDVRKIL MGPCELPETL GLILTLQRLA LDYYYENEID KLLHCIY-DD DDSDYDDKDL NLVSLRFYLL
Zm00037ab419380_NC358       MAPPPPAL-- -----AEETA AFHPSLWGDF FLTYQPPTAP QQACMKERAE VLREDVRKIL MGPCELPETL GLILTLQRLA LDYYYENEID KLLHCIY-DD DDSDYDDKDL NLVSLRFYLL
Zm00038ab425330_Oh7B        MAPPPPAL-- -----AEETA AFHPSLWGDF FLTYQPPTAP QQACMKERAE VLREDVRKIL MGPCELPETL GLILTLQRLA LDYYYENEID KLLHCIY-DD DDSDYDDKDL NLVSLRFYLL
Zm00041ab432750_Tx303       MAPPPPALLA GSSKAAEETT SFHPSLWGDF FLTYQPPTAP QQACMKERAE VLREDVRKIL MGPCELPETL GLILTLQRLA LDYYYEKEID KLLHCIYNDD DDSDYDDKDL NLVSLRFYLL
Zm00036ab427620_NC350       MAPPPPALLA GSSKAAEETT SFHPSLWGDF FLTYQPPTAP QQACMKERAE VLREDVRKIL MGPCELPETL GLILTLQRLA LDYYYEKEID KLFHCIYNDD DDSDYDDKDL NLVSLRFYLL

                            ....|....| ....|....| ....|....| ....|....| ....|....| ....|....| ....|....| ....|....| ....|....| ....|....| ....|....| ....|....|
                                 125        135        145        155        165        175        185        195        205        215        225        235
Zm00004b038743_W22          RKNGYDVSSD VFLDFKTEEG SFAYAD-TAS LLSLYNAAYL RKHGEEVLDE AISFARRRC- LQAGI----- ---------- ----LGTPLF RRVGILEARN YIPIYEKEAA RNDAVLELAK
Zm00001d024477_B73          RKNGYDVSSD VFLVFKTEEG SFAYADDTAS LLSLYNAAYL RKHGEEVLDE AISFARRRC- LEAGI----- ---------- ----LRTPLF RRVGILEARN YIPIYEKEAA RNDAVLELAK
Zm00039ab424100_Oh43        RKNGYDVSSD VFLVFKTEEG SFAYADDTAS LLSLYNAAYL RKHGEEVLDE AISFARRRC- LEAGI----- ---------- ----LRTPLF RRVGILEARN YIPIYEKEAA RNDAVLELAK
Zm00022ab421380_CML228      RKNGYDVSSD VFLDFKTEEG SFAYAD-TAS LLSLYNAAYL RKHGEEVLDE AISFARRRC- LEAGI----- ---------- ----LRTPLF RRVGILEARN YIPIYEKEAA RNDAVLELAK
Zm00023ab427130_CML247      RKNGYDVSSD VFLDFKTEEG SFAYADDTAS LLSLYNAAYL RKHGEEVLDE AISFARRRC- LEAGI----- ---------- ----LRTPLF RRVGILEARN YIPIYEKEAA RNDAVLELAK
Zm00024ab424620_CML277      RKNGYDVSSD VFLDFKTEEG SFAYADDTAS LLSLYNAAYL RKHGEEVLDE AISFARRRC- LEAGI----- ---------- ----LRTPLF RRVGILEARN YIPIYEKEAA RNDAVLELAK
Zm00026ab417460_CML333      RKNGYDVSSD VFLDFKTEEG SFAYAD-TAS LLSLYNAAYL RKHGEEVLDE AISFARRRC- LEAGI----- ---------- ----LRTPLF RRVGILEARN YIPIYEKEAA RNDAVLELAK
Zm00019ab401530_CML52       RKNGYDVSSD VFLDFKTEEG SFAYAD-TAS LLSLYNAAYL RKHGEEVLDE AISFARRRC- LEAGI----- ---------- ----LRTPLF RRVGILEARN YIPIYEKEAA RNDAVLELAK
Zm00020ab421560_CML69       RKNGYDVSSD VFLDFKTEEG SFAYADDTAS LLSLYNAAYL RKHGEEVLDE AISFARRRC- LEAGI----- ---------- ----LRTPLF RRVGILEARN YIPIYEKEAA RNDAVLELAK
Zm00030ab419080_Ki11        RKNGYDVSSD VFLDFKTEEG SFAYADDTAS LLSLYNAAYL RKHGEEVLDE AISFARQRC- LEAGIVLLEH TESPFA-REP VSSSLGTPLF RRVGILEARN YIPIYEKEAA RNDAVLELAK
Zm00034ab441680_Mo18W       RKNGYDVSSD VFLVFKTEEG SFAYADDTAS LLSLYNAAYL RKHGEEVLDE AISFARRRC- LEAGI----- ---------- ----LRTPLF RRVGILEARN YIPIYEKEAA RNDAVLELAK
Zm00037ab419380_NC358       RKNGYDVSSD VFLDFKTEEG SFAYADDTAS LLSLYNAAYL RKHGEEVLDE AISFARRRC- LEAGI----- ---------- ----LRTPLF RRVGILEARN YIPIYEKEAA RNDAVLELAK
Zm00038ab425330_Oh7B        RKNGYDVSSD VFLDFKTEEG SFAYAD-TAS LLSLYNAAYL RKHGEEVLDE AISFARRRC- LQAGI----- ---------- ----LGTPLF RRVGILEARN YIPIYEKEAA RNDAVLELAK
Zm00041ab432750_Tx303       RKNGYDVSSD VFLDFKTEEG SFAYADDTAS LLSLYNAAYL RKHGEEVLDE AISFARRRC- LEAGI----- ---------- ----LRTPLF RRVGILEARN YIPIYEKEAA RNDAVLELAK
Zm00036ab427620_NC350       RKNGYDVSSD VFLDFKTEEG SFAYAD-TAS LLSLYNAAYL RKHGEEVLDE AISFARRRC- LQAGI----- ---------- ----LGTPLF RRVGILEARN YIPIYEKEAA RNDAVLELAK

                                                                RXR
                            ....|....| ....|....| ....|....| ....|....| ....|....| ....|....| ....|....| ....|....| ....|....| ....|....| ....|....| ....|....|
                                 245        255        265        275        285        295        305        315        325        335        345        355
Zm00004b038743_W22          LNFNLQQIAF CEELKHCTLW WKDFLAKSKM SFVRDRIVEV YFWMNGACYD PPYSRSRIIL TKITSLVTII DDMFDTYGTT EDCTKFHEAI GRWDESALPL LPQYMKGFYL FQLETFRSFE
Zm00001d024477_B73          LNFNLQQIAF CEELKHCTLW WKDFLAKSKM AFVRDRIVEV YFWMNGACYD PPYSRSRIIL TKITSLVTII DDMFDTYGTT EDCTKFHEAI GRWDESALPL LPQYMKGFYL FQLETFRSFE
Zm00039ab424100_Oh43        LNFNLQQIAF CEELKHCTLW WKDFLAKSKM AFVRDRIVEV YFWMNGACYD PPYSRSRIIL TKITSLVTII DDMFDTYGTT EDCTKFHEAI GRWDESALPL LPQYMKGFYL FQLETFRSFE
Zm00022ab421380_CML228      LNFNLQQIAF CEELKHCTLW WKDFLAKSKM AFVRDRIVEV YFWMNGACYD PPYSRSRIIL TKITSLVTII DDMFDTYGTT EDCTKFHEAI GRWDESALPL LPQYMKGFYL FQLETFRSFE
Zm00023ab427130_CML247      LNFNLQQIAF CEELKHCTLW WKDFLAKSKM AFVRDRIVEV YFWMNGACYD PPYSRSRIIL TKITSLVTII DDMFDTYGTT EDCTKFHEAI GRWDESALPL LPQYMKGFYL FQLETFRSFE
Zm00024ab424620_CML277      LNFNLQQIAF CEELKHCTLW WKDFLAKSKM AFVRDRIVEV YFWMNGACYD PPYSRSRIIL TKITSLVTII DDMFDTYGTT EDCTKFHEAI GRWDESALPL LPQYMKGFYL FQLETFRSFE
Zm00026ab417460_CML333      LNFNLQQIAF CEELKHCTLW WKDFLAKSKM AFVRDRIVEV YFWMNGACYD PPYSRSRIIL TKITSLVTII DDMFDTYGTT EDCTKFHEAI GRWDESALPL LPQYMKGFYL FQLETFRSFE
Zm00019ab401530_CML52       LNFNLQQIAF CEELKHCTLW WKDFLAKSKM AFVRDRIVEV YFWMNGACYD PPYSRSRIIL TKITSLVTII DDMFDTYGTT EDCTKFHEAI GRWDESALPL LPQYMKGFYL FQLETFRSFE
Zm00020ab421560_CML69       LNFNLQQIAF CEELKHCTLW WKDFLAKSKM AFVRDRIVEV YFWMNGACYD PPYSRSRIIL TKITSLVTII DDMFDTYGTT EDCTKFHEAI GRWDESALPL LPQYMKGFYL FQLETFRSFE
Zm00030ab419080_Ki11        LNFNLQQIAF CEELKHCTLW WKDFLAKSKM AFVRDRIVEV YFWMNGACYD PPYSRSRIIL TKITSLVTII DDMFDTYGTT EDCTKFHEAI GRWDESALPL LPQYMKGFYL FQLETFRSFE
Zm00034ab441680_Mo18W       LNFNLQQIAF CEELKHCTLW WKDFLAKSKM AFVRDRIVEV YFWMNGACYD PPYSRSRIIL TKITSLVTII DDMFDTYGTT EDCTKFHEAI GRWDESALPL LPQYMKGFYL FQLETFRSFE
Zm00037ab419380_NC358       LNFNLQQIAF CEELKHCTLW WKDFLAKSKM AFVRDRIVEV YFWMNGACYD PPYSRSRIIL TKITSLVTII DDMFDTYGTT EDCTKFHEAI GRWDESALPL LPQYMKGFYL FQLETFRSFE
Zm00038ab425330_Oh7B        LNFNLQQIAF CEELKHCTLW WKDFLAKSKM SFVRDRIVEV YFWMNGACYD PPYSRSRIIL TKITSLVTII DDMFDTYGTT EDCTKFHEAI GRWDESALPL LPQYMKGFYL FQLETFRSFE
Zm00041ab432750_Tx303       LNFNLQQIAF CEELKHCTLW WKDFLAKSKM AFVRDRIVEV YFWMNGACYD PPYSRSRIIL TKITSLVTII DDMFDTYGTT EDCTKFHEAI GRWDESALPL LPQYMKGFYL FQLETFRSFE
Zm00036ab427620_NC350       LNFNLQQIAF CEELKHCTLW WKDFLAKSKM AFVRDRIVEV YFWMNGACYD PPYSRSRIIL TKITSLVTII DDMFDTYGTT EDCTKFHEAI GRWDESALPL LPQYMKGFYL FQLETFRSFE

                                                                                                                         NSE/DTE
                            ....|....| ....|....| ....|....| ....|....| ....|....| ....|....| ....|....| ....|....| ....|....| ....|....| ....|....| ....|....|
                                 365        375        385        395        405        415        425        435        445        455        465        475
Zm00004b038743_W22          DELGPENSYR -------MER LVQQYYREIK WRDEDYVPKT MTEHLQVSME SIGSVALACA AYVGMDDVIT KETLDWVLSY PQFLTTYGTI VRLSNDVVST KREQTKDHSP STVHCYMKDH
Zm00001d024477_B73          DELGPENSYR -------MER LVQQYYREIK WRDENYVPKT MTEHLQVSME SIGSVALACA AYVGMGDVIT KETLDWVLSY PQFLTTYG-I VRLSNDVVST KREQTKDHSP STVHCYMKEH
Zm00039ab424100_Oh43        DELGPENSYR VHYLKKSMER LVQQYYREIK WRDENYVPKT MTEHLQVSME SIGSVALACA AYVGMGDVIT KETLDWVLSY PQFLTTYG-I VRLSNDVVST KREQTKDHSP STVHCYMKEH
Zm00022ab421380_CML228      DELGPENSYR VHYLKKSMER LVQQYYREIK WRDEDYVPKT MTEHLQVSME SIGSVALACA AYVGMGDVIT KETLDWVLSY PQFLTTYGTI VRLSNDVVST KREQTKDHSP STVHCYMKEH
Zm00023ab427130_CML247      DELGPENSYR VHYLKKSMER LVQQYYREIK WRDEDYVPKT MTEHLQVSME SIGSVALACA AYVGMGDVIT KETLDWVLSY PQFLTTYGTI VRLSNDVVST KREQTKDHSP STVHCYMKEH
Zm00024ab424620_CML277      DELGPENSYR VHYLKKSMER LVQQYYREIK WRDEDYVPKT MTEHLQVSME SIGSVALACA AYVGMGDVIT KETLDWVLSY PQFLTTYGTI VRLSNDVVST KREQTKDHSP STVHCYMKEH
Zm00026ab417460_CML333      DELGPENSYR VHYLKKSMER LVQQYYREIK WRDEDYVPKT MTEHLQVSME SIGSVALACA AYVGMGDVIT KETLDWVLSY PQFLTTYGTI VRLSNDVVST KREQTKDHSP STVHCYMKEH
Zm00019ab401530_CML52       DELGPENSYR VHYLKKSMER LVQQYYREIK WRDEDYVPKT MTEHLQVSME SIGSVALACA AYVGMGDVIT KETLDWVLSY PQFLTTYGTI VRLSNDVVST KREQTKDHSP STVHCYMKEH
Zm00020ab421560_CML69       DELGPENSYR VHYLKKSMER LVQQYYREIK WRDEDYVPKT MTEHLQVSME SIGSVALACA AYVGMGDVIT KETLDWVLSY PQFLTTYGTI VRLSNDVVST KREQTKDHSP STVHCYMKEH
Zm00030ab419080_Ki11        DELGPENSYR VHYLKKSMER LVQQYYREIK WRDENYVPKT MTEHLQVSME SIGSVALACA AYVGMGDVIT KETLDWVLSY PQFLTTYGTI VRLSNDVVST KREQTKDHSP STVHCYMKEH
Zm00034ab441680_Mo18W       DELGPENSYR VHYLKKSMER LVQQYYREIK WRDENYVPKT MTEHLQVSME SIGSVALACA AYVGMGDVIT KETLDWVLSY PQFLTTYG-I VRLSNDVVST KREQTKDHSP STVHCYMKEH
Zm00037ab419380_NC358       DELGPENSYR VHYLKKSMER LVQQYYREIK WRDEDYVPKT MTEHLQVSME SIGSVALACA AYVGMGDVIT KETLDWVLSY PQFLTTYGTI VRLSNDVVST KREQTKDHSP STVHCYMKEH
Zm00038ab425330_Oh7B        DELGPENSYR VHYLKKSMER LVQQYYREIK WRDEDYVPKT MTEHLQVSME SIGSVALACA AYVGMDDVIT KETLDWVLSY PQFLTTYGTI VRLSNDVVST KREQTKDHSP STVHCYMKDH
Zm00041ab432750_Tx303       DELGPENSYR VHYLKKSMER LVQQYYREIK WRDEDYVPKT MTEHLQVSME SIGSVALACA AYVGMGDVIT KETLDWVLSY PQFLTTYGTI VRLSNDVVST KREQTKDHSP STVHCYMKEH
Zm00036ab427620_NC350       DELGPENSYR VHYLKKSMER LVQQYYREIK WRDEDYVPKT MTEHLQVSME SIGSVALACA AYVGMDDVIT KETLDWVLSY PQFLTTYGTI VRLSNDVVST KREQTKDHSP STVHCYMKDH

                            ....|....| ....|....| ....|....| ....|....| ....|....| ....|....| ....|....| ....|..
                                 485        495        505        515        525        535        545        555
Zm00004b038743_W22          GTTMDDACEK IKELIEDSWK DMLEQSLALK GLPKAVPRTV FDFLRTADNM YKNCDAFTSS EVLREMIRLL FVEPIPE
Zm00001d024477_B73          GTTMDDACEK IKELIEDSWK DMLQQSLALK GLPKAVPRTV FDFLRTADNM YKNCDAFTSS EVLREMIRLL FVEPIPE
Zm00039ab424100_Oh43        GTTMDDACEK IKELIEDSWK DMLQQSLALK GLPKAVPRTV FDFLRTADNM YKNCDAFTSS EVLREMIRLL FVEPIPE
Zm00022ab421380_CML228      GTTMDDACEK IKELIEDSWK DMLEQSLALK GLPKAVPRTV FDFLRTADNM YKNCDAFTSS EVLREMIRLL FVEPIPE
Zm00023ab427130_CML247      GTTMDDACEK IKELIEDSWK DMLQQSLALK GLPKAVPRTV FDFLRTADNM YKNCDAFTSS EVLREMIRLL FVEPIPE
Zm00024ab424620_CML277      GTTMDDACEK IKELIEDSWK DMLQQSLALK GLPKAVPRTV FDFLRTADNM YKNCDAFTSS EVLREMIRLL FVEPIPE
Zm00026ab417460_CML333      GTTMDDACEK IKELIEDSWK DMLEQSLALK GLPKAVPRTV FDFLRTADNM YKNCDAFTSS EVLREMIRLL FVEPIPE
Zm00019ab401530_CML52       GTTMDDACEK IKELIEDSWK DMLEQSLALK GLPKAVPRTV FDFLRTADNM YKNCDAFTSS EVLREMIRLL FVEPIPE
Zm00020ab421560_CML69       GTTMDDACEK IKELIEDSWK DMLQQSLALK GLPKAVPRTV FDFLRTADNM YKNCDAFTSS EVLREMIRLL FVEPIPE
Zm00030ab419080_Ki11        GTTMDDACEK IKELIEDSWK DMLEQSLALK GLPKAVPRTV FDFLRTADNM YKNCDAFTSS EVLREMIRLL FVEPIPE
Zm00034ab441680_Mo18W       GTTMDDACEK IKELIEDSWK DMLQQSLALK GLPKAVPRTV FDFLRTADNM YKNCDAFTSS EVLREMIRLL FVEPIPE
Zm00037ab419380_NC358       GTTMDDACEK IKELIEDSWK DMLQQSLALK GLPKAVPRTV FDFLRTADNM YKNCDAFTSS EVLREMIRLL FVEPIPE
Zm00038ab425330_Oh7B        GTTMDDACEK IKELIEDSWK DMLEQSLALK GLPKAVPRTV FDFLRTADNM YKNCDAFTSS EVLREMIRLL FVEPIPE
Zm00041ab432750_Tx303       GTTMDDACEK IKELIEDSWK DMLQQSLALK GLPKAVPRTV FDFLRTADNM YKNCDAFTSS EVLREMIRLL FVEPIPE
Zm00036ab427620_NC350       GTTMDDACEK IKELIEDSWK DMLEQSLALK GLPKAVPRTV FDFLRTADNM YKNCDAFTSS EVLREMIRLL FVEPISE
```

Figure S3. ZmTPS9 amino acid sequence comparison across selected maize inbred lines. The alignment of predicted amino acid sequences encoded by maize inbred lines was computed with the program MEGA7 (www.megasoftware.net) and the MUSCLE algorithm. The visualization was performed with the program BIOEDIT (http://www.mbio.ncsu.edu/BioEdit). A deletion of an active-site residue is indicated by a red triangle. The RxR and NSE/DTE motives are marked with red boxes.

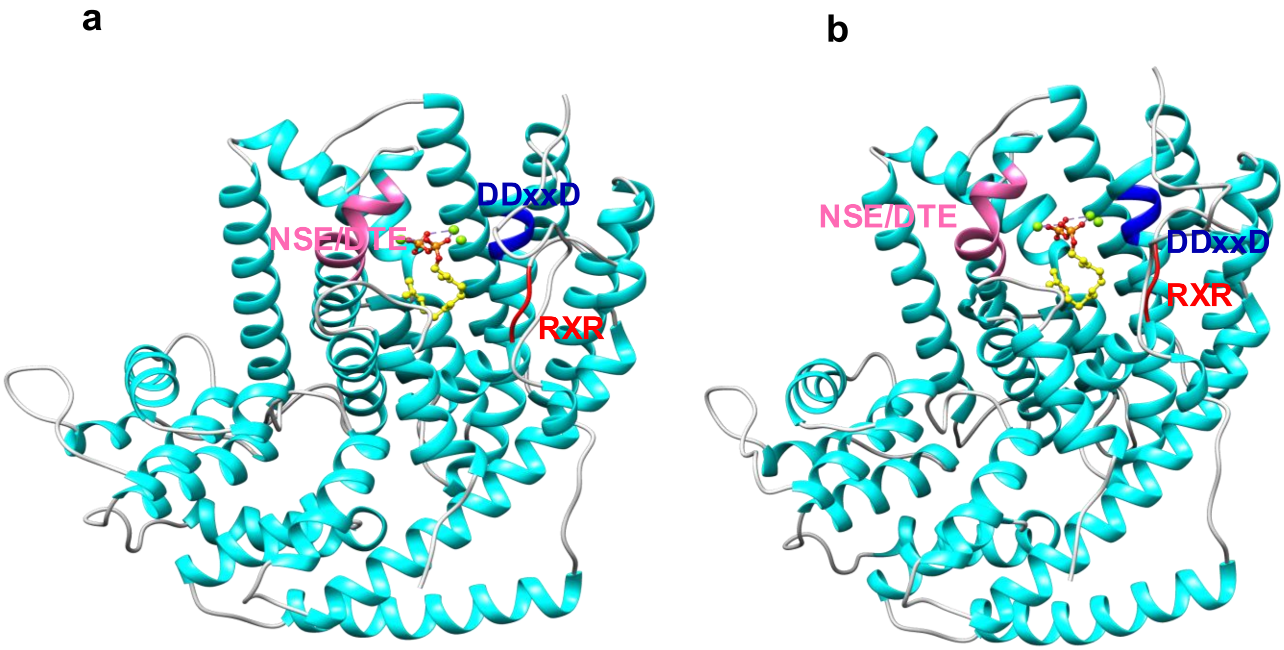

Figure S4. Three-dimensional model of the maize terpene synthase ZmTPS9. a, 3D model of ZmTPS9-W22. **b**, Predicted 3D model of ZmTPS9-B73. The homology-based models were generated with Modeller v10.3 using multi-template modeling based on the following template structures: 7CJY, 3G4D, 5IK0, 5EAT, 5ZZJ, and 6A1D. The substrate analog, FPP, is shown as a yellow stick model, and the two Mg^{2+} atoms are represented as magenta spheres.

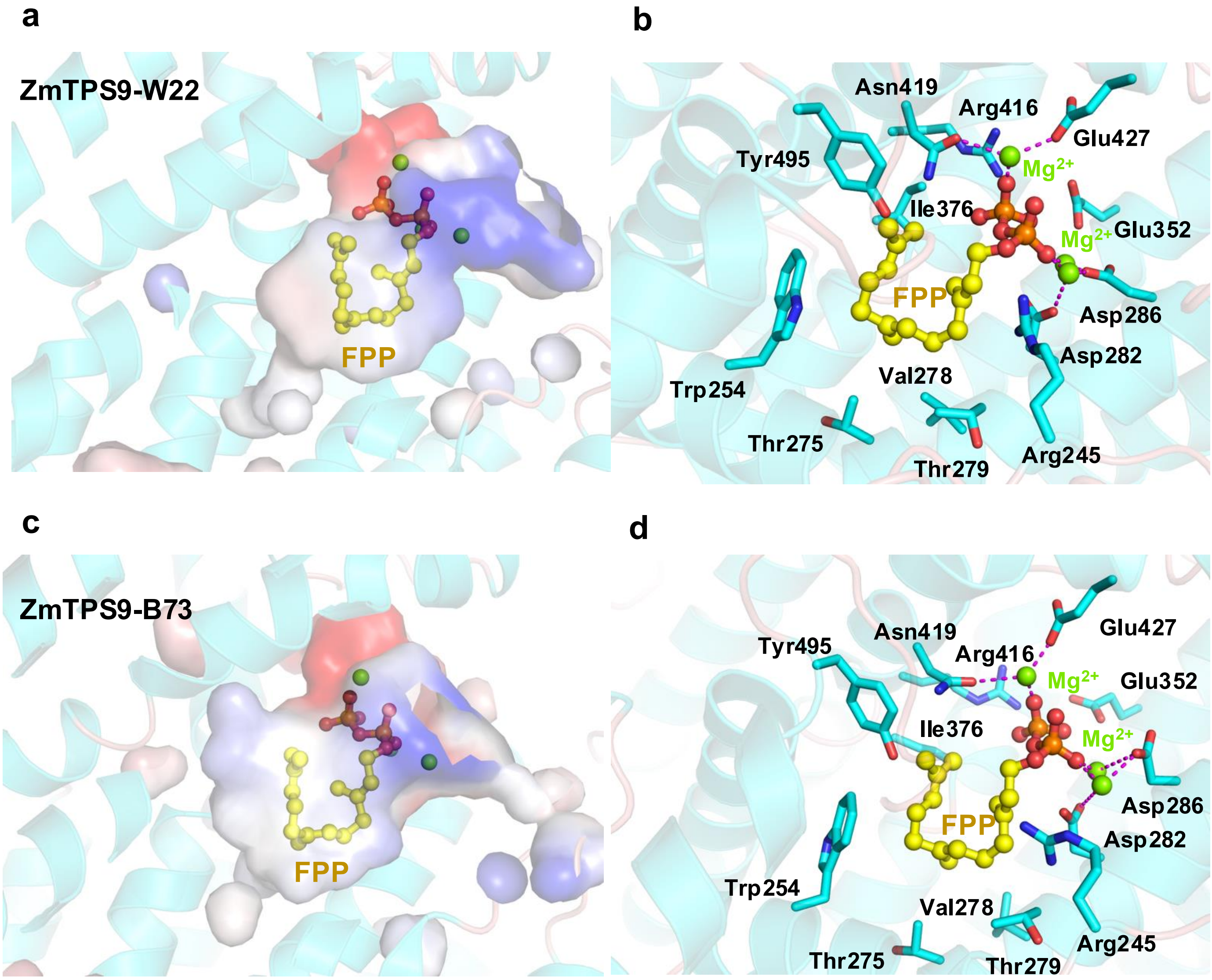

Figure S5. Substrate-binding pocket and FPP docking in ZmTPS9. a,c, Surface electrostatic representation of the hydrophobic active site pocket of ZmTPS9-W22 (a) and ZmTPS9-B73 (c) with bound FPP. **b,d,** Interaction of amino acid residues with FPP in the binding pockets of ZmTPS9-W22 (b) and ZmTPS9-B73 (d). The bound FPP is depicted as a ball-and-stick model. The residues in the active site of TPS9 are shown.

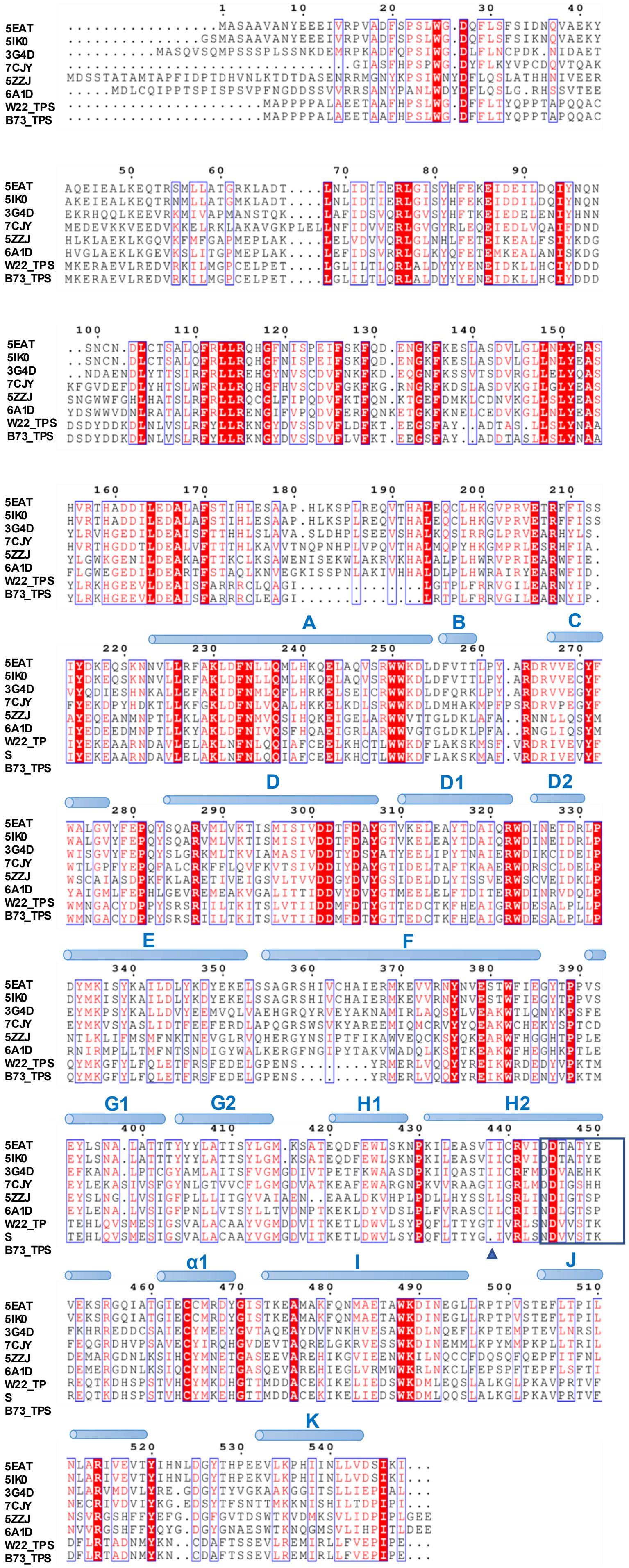

Figure S6. Amino acid sequence comparison of ZmTPS9 and other plant TPSs. Comparison of amino acid sequences of 7CJY, 3G4D, 5IK0, 5EAT, 5ZZJ, and 6A1D. Helixes A to K (in blue) are labeled on the sequence.

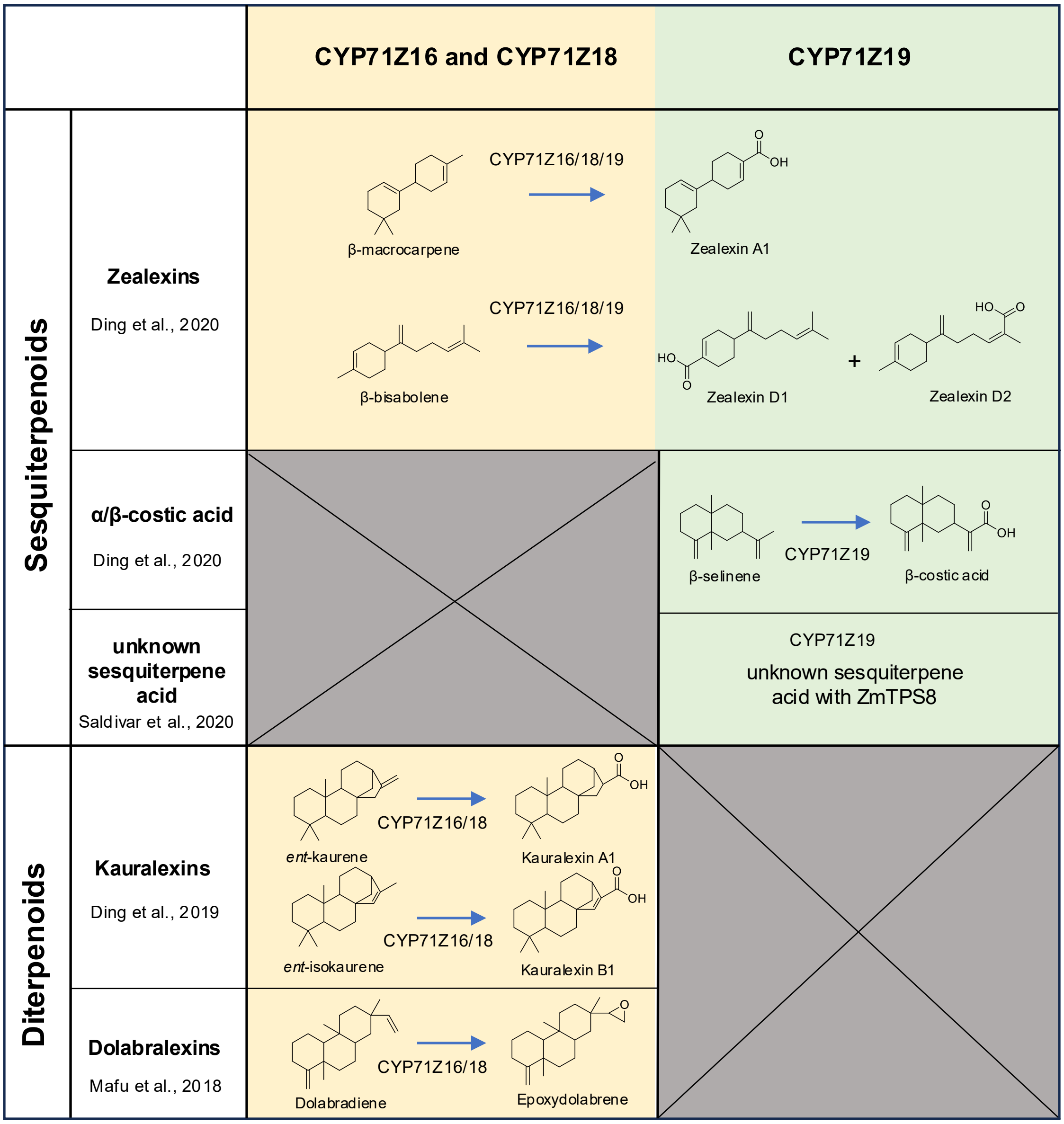

Figure S7. Functional overview of three ZmCYP71Z P450 enzymes in maize terpenoid metabolism. ZmCYP71Z19 functions exclusively in the sesquiterpenoid pathway, while ZmCYP71Z16 and ZmCYP71Z18 participate in both sesquiterpenoid and diterpenoid biosynthetic routes.

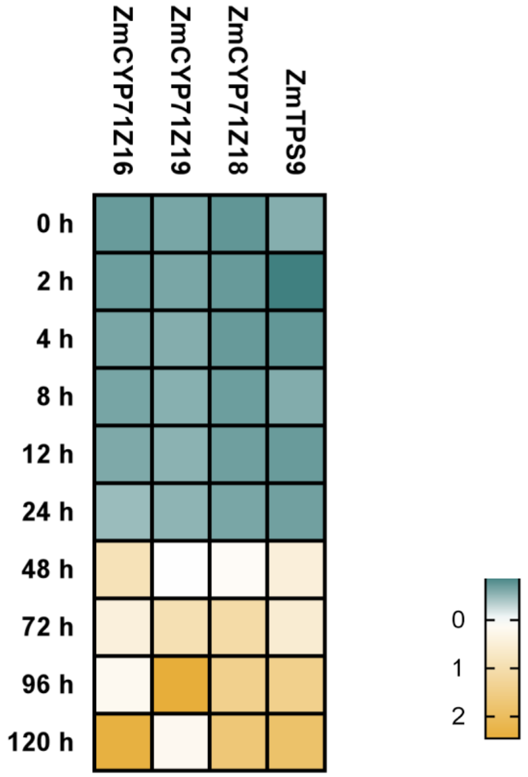

Figure S8. Heatmap depicting temporal protein expression dynamics in W22 stem tissues following elicitation with heat-killed *Fusarium venenatum*. Protein abundance was quantified based on unique peptides mapped to predicted gene models from the B73 RefGen v4 genome. Expression values were z-score normalized prior to downstream analysis.

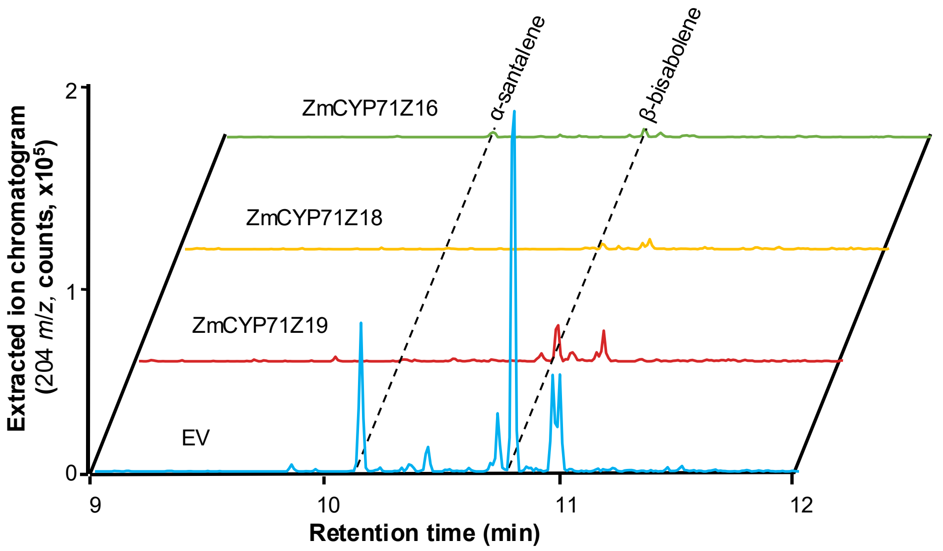

Figure S9. GC–MS analysis of transiently transformed *N. benthamiana* leaves co-expressing *ZmTPS9* with individual *ZmCYP71Z* P450 genes (*ZmCYP71Z16, ZmCYP71Z18, and ZmCYP71Z19*) or with the empty vector (EV) control. Co-expression of ZmTPS9 with each P450 enzyme resulted in a marked reduction of ZmTPS9-derived olefin products, indicating enzymatic conversion. The same GC-MS data files analyzed in Figure 5a were used for this comparison.

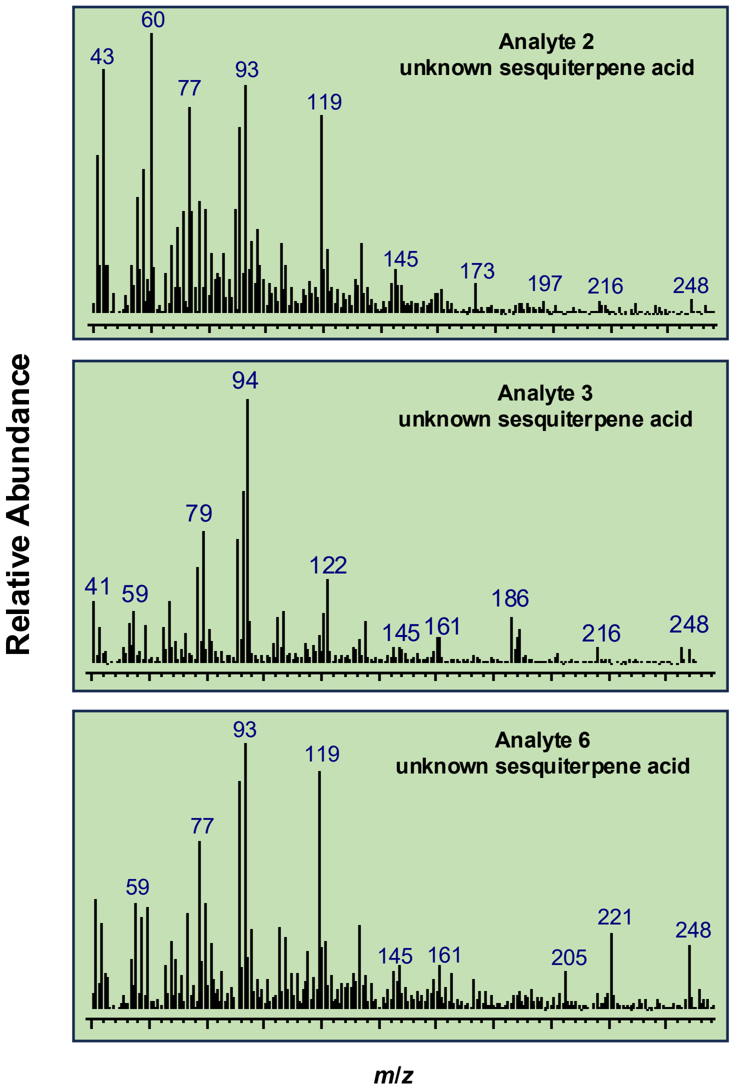

Figure S10. EI mass spectra of unidentified sesquiterpene acids detected in *N. benthamiana* leaf tissues transiently co-expressing *ZmTPS9* with individual P450 genes (*ZmCYP71Z16, ZmCYP71Z18, or ZmCYP71Z19*), as well as with the empty vector (EV) control. Corresponding GC-MS chromatograms are shown in Figure 5a.

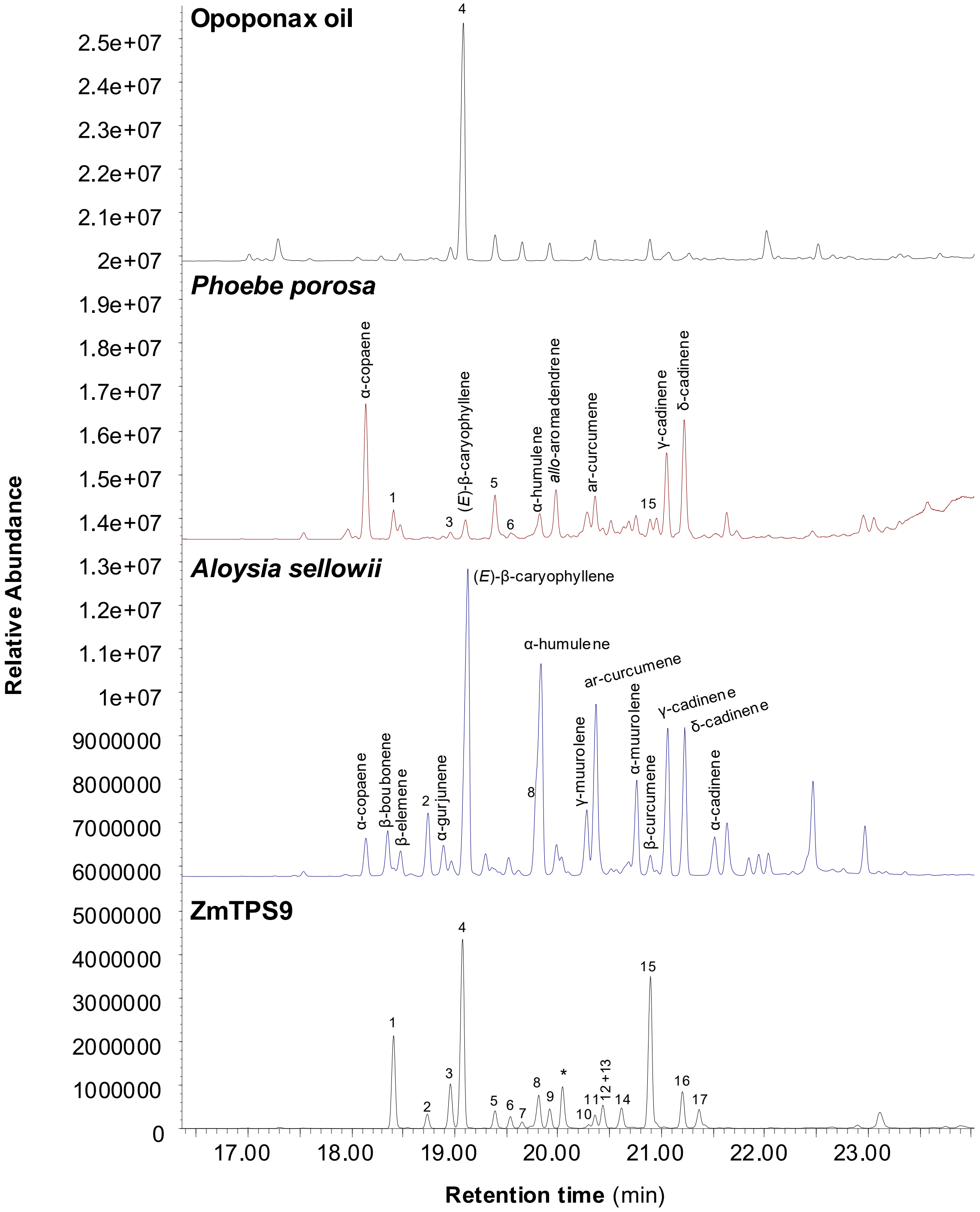

Figure S11. Identification of ZmTPS9 sesquiterpene products. Opoponax oil and essential oils from *Aloysia sellowii* (hydrocarbon fraction) and *Phoebe porosa* were used as reference standards for the identification of ZmTPS9 products. Essential oils with peak assignments were kindly provided by Wilfried A, König, Hamburg, Germany. 1, 7-*epi*-sesquithujene; 2, sesquithujene; 3, *cis*-α-bergamotene; 4, α-santalene; 5, *trans*-α-bergamotene; 6, sesquisabinene A; 7, *epi*-β-santalene; 8, sesquisabinene B; 9, β-santalene; 10, γ-curcumene; 11, *ar*-curcumene; 12, unidentified sesquiterpene; 13, unidentified sesquiterpene; 14, α-zingiberene; 15, β-bisabolene; 16, β-sesquiphellandrene; 17, *trans*-γ-bisabolene; * contaminants. Other ZmTPS9-derived products not present in the three oils were identified through comparison with reference mass spectra from the NIST and Wiley spectral databases.

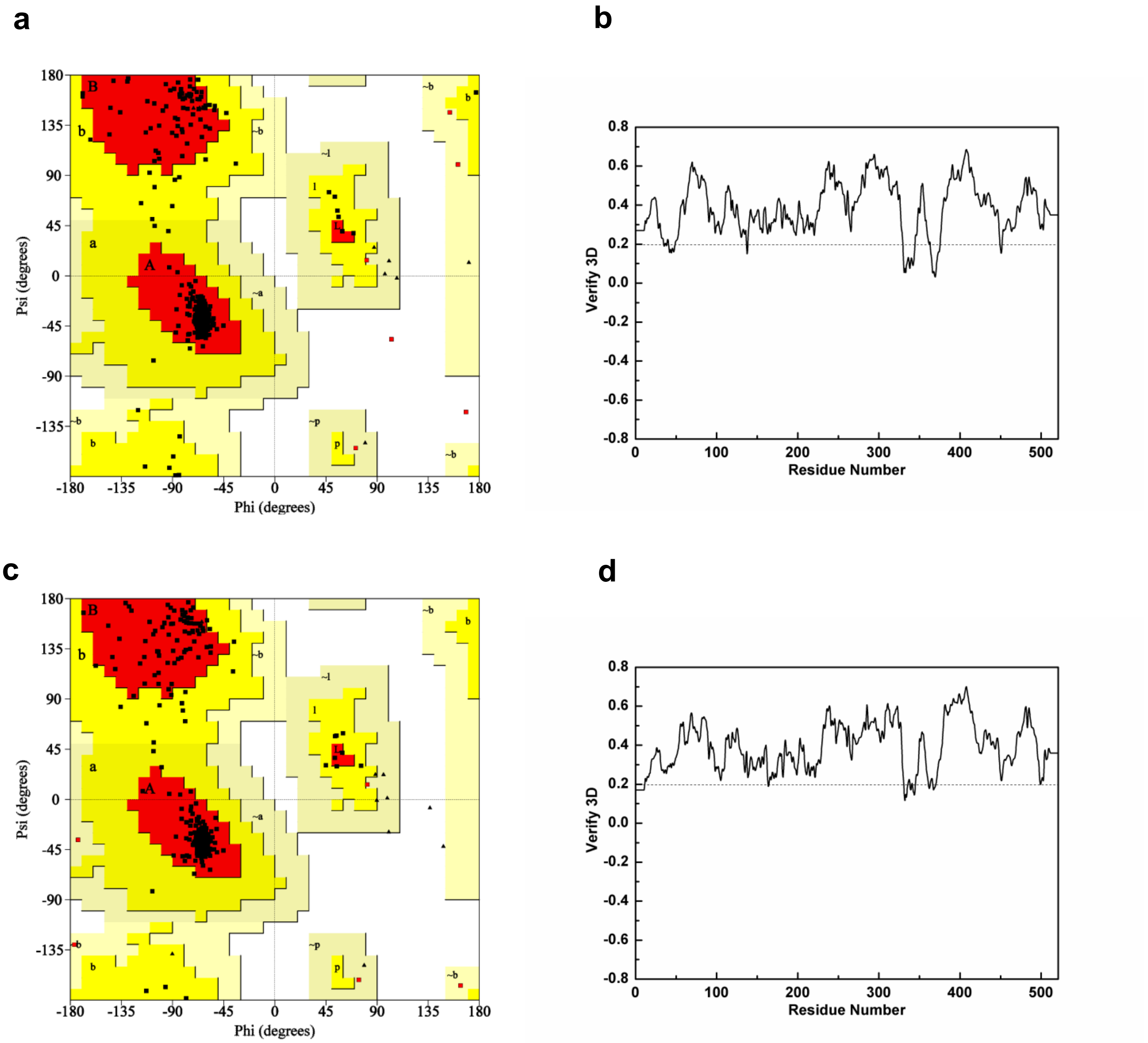

Figure S12. Ramachandran plots and Verify3D scores of TPS9 models. Ramachandran plot (a and c) and Verify 3D score (b and d) of TPS9 active protein and mutant models.
